# Impact of remotely generated eddies on plume dispersion at abyssal mining sites in the Pacific

**DOI:** 10.1038/s41598-017-16912-2

**Published:** 2017-12-05

**Authors:** Dmitry Aleynik, Mark E. Inall, Andrew Dale, Annemiek Vink

**Affiliations:** 1 SAMS, Scottish Association for Marine Science, Scottish Marine Institute, Oban, PA37 1QA UK; 2 BGR, Bundesanstalt für Geowissenschaften und Rohstoffe, Stilleweg 2, 30655 Hannover, Germany; 3 University of Edinburgh, School of Geosciences, Edinburgh, EH9 3FE UK

## Abstract

Proposed harvesting of polymetallic nodules in the Central Tropical Pacific will generate plumes of suspended sediment which are anticipated to be ecologically harmful. While the deep sea is low in energy, it also can be highly turbulent, since the vertical density gradient which suppresses turbulence is weak. The ability to predict the impact of deep plumes is limited by scarcity of *in-situ* observations. Our observations show that the low-energy environment more than four kilometres below the surface ultimately becomes an order of magnitude more energetic for periods of weeks in response to the passage of mesoscale eddies. The source of these eddies is remote in time and space, here identified as the Central American Gap Winds. Abyssal current variability is controlled by comparable contributions from tides, surface winds and passing eddies. During eddy-induced elevated flow periods mining-related plumes, potentially supplemented by natural sediment resuspension, are expected to spread and disperse more widely and rapidly. Predictions are given of the timing, location and scales of impact.

## Introduction

**Figure 1:**
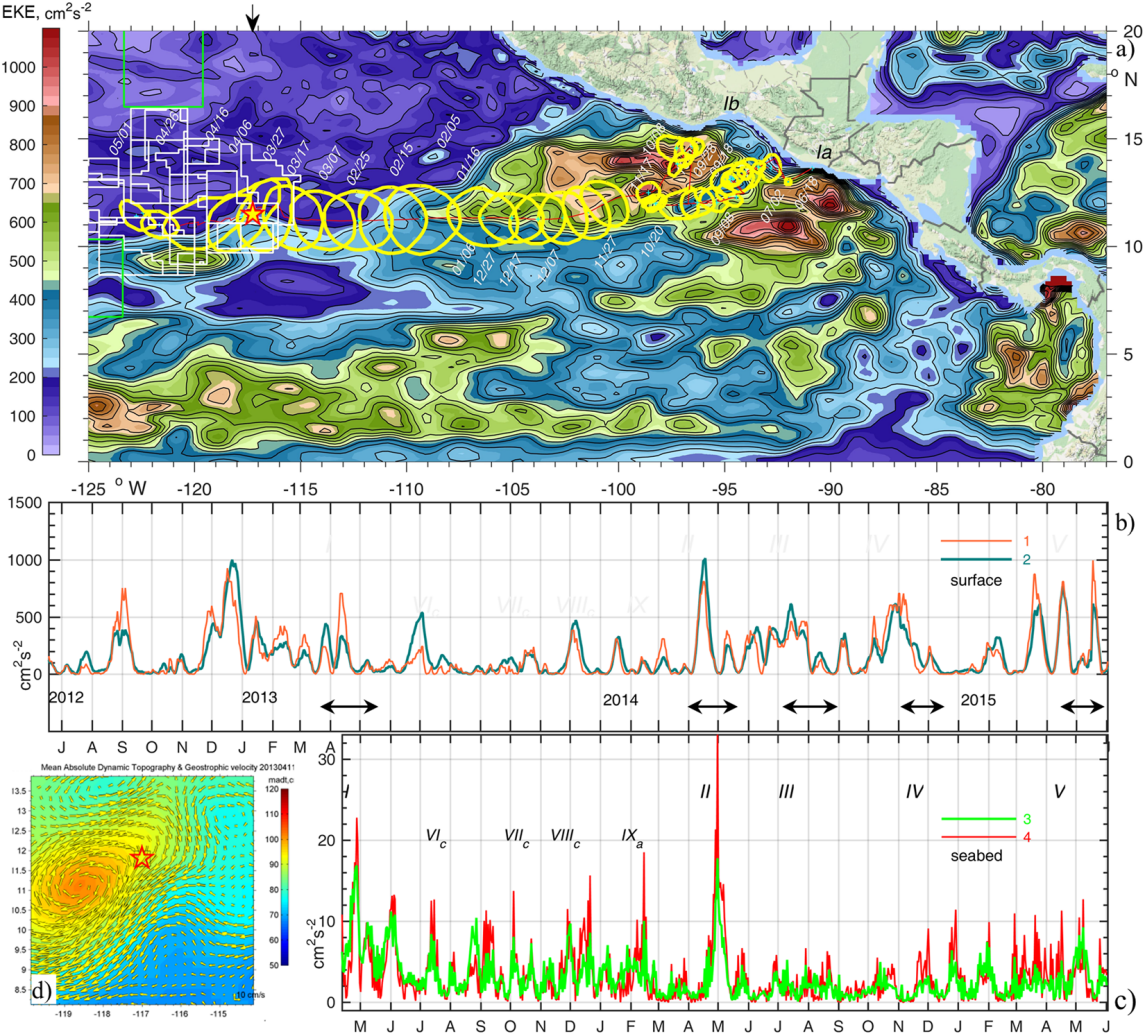
(**a**) The track of a mesoscale eddy (I) over 318 days from the coast towards the CCZ (licence areas shown with white and APEIs with green lines) and the BGR moorings site (star). Yellow lines encircle a local maximum (>80 cm) sea surface height (SSH) anomaly (AVISO^31^) at 10-day intervals. Colours reflect mean surface Eddy Kinetic Energy (EKE) over the period 2012.06.18-2013.05.01. (**b**) Timeseries of EKE at the sea-surface in the nearest () and the averaged over adjacent four grid points around the moorings site (). (**c**) EKE series in a layer 15–20 mab averaged over all three moorings () and at the northern site (). Here EKE = *0.5*·(*u*′^*2*^ + *v*′^2^), and *u*′, *v*′ are the deviations of *u,v* velocities from the mean *ū*, $\bar{v}$ averaged over the shown 3 and 2 years respectively. Eddies arrivals are indicated with black arrows and latin numbers I-V, IX (anticyclonic) and *VI*_*c*_*-VIII*_*c*_ (cyclonic). (**d**) Inset shows SSH anomaly (colours) and geostrophic currents (arrows) on the date of moorings deployment (2013.04.11). Figure was plotted using MATLAB R2015b (http://www.mathworks.com/).The map in this figure was queried from Google Static Map APIs (http://code.google.com/apis/maps/) using Get_google_map mapping package version 1.4 (https://uk.mathworks.com/matlabcentral/fileexchange/24113-get-google-map)

Interest in deep-sea mining is driven by growing demand for metals such as nickel, cobalt, copper and especially rare earth elements. Exploration activities focus on polymetallic crusts on seamounts, polymetallic nodules on abyssal plains and massive sulphides on mid-ocean ridges. As in Klondike times, the past six years of Blue Growth Rush has tripled the number of concessions licenced by the International Seabed Authority (ISA) to a total of 25 for exploration “in the Heart of the Sea”^1^ – the Central Tropical Pacific. In 2011, in accordance with the concept of sustainable development and the “common heritage of mankind”, nine Areas of Particular Environmental Interest (APEI)^2^ surrounding the nodule concessions in the Clarion-Clipperton Zone (CCZ) were allocated for protection purposes (Fig. 1a). The long-lasting (decadal) effects of mining, including impacts resulting directly from removal of bottom sediment and nodule habitat and indirectly from rising and settling plumes of Suspended Particulate Matter (SPM), were quickly recognised^3^. These include negative effects on community structure and biodiversity in all faunal size classes^4,5^. The need for regional environmental baseline assessment and comprehensive spatial management planning^6^ including the allocation of marine environmental protected areas beyond the proposed mining sites (e.g. APEIs) as well as within concession areas (e.g. Preservation Reference Zones) is an urgent requirement for the protection of this extremely diverse, unique and largest abyssal benthic community on Earth^7,8^. Several exploration contracts expired in 2016, however, the regulations and standard contract terms for exploitation of mineral resources in the areas beyond national jurisdiction are currently still under development^9^. Hence, tools to assist spatial planning processes and to develop mitigation measures to minimize impacts of mining, as proposed in this paper, are urgently required. Here, we focus our attention on modelling the fate and spreading potential of dissolved and particulate material suspended in deep-sea mining scenarios based on the analysis of the natural variability of hydrographic conditions at the sea-surface and near the seafloor in the CCZ.

Despite the well-known fact that surface-generated, full-depth eddies are important contributors for the material transport to the interior ocean from continental margins^10,11^ and hydrothermal vent systems of mid-ocean ridges such as East Pacific Rise^12^, little is known of their near-seabed effects over abyssal plains, particularly with respect to local resuspension. The size of long-lived coherent mesoscale eddies follows the first baroclinic Rossby radius of deformation, which varies between 70 and 100 km at 10–15°N in Pacific^13^. Extreme examples of strong deep-ocean flow include turbidity currents in canyons of continental slopes^14^ and so-called benthic storms^15^. In two accounts^16,17^ of western ocean margins, a synoptic flow-field related to the propagation of large (mesoscale) features was coherent over the full ocean depth. In the Central Tropical Pacific, the influence of surface eddies on the currents at abyssal depths has also been noted^18,19^.

Wind energy transfer by internal gravity waves of near the inertial (Coriolis) frequency *f* into the deep ocean is smaller than, but comparable to, overall barotropic tidal dissipation. Furthermore the direct dynamical impact of a fast-moving hurricane may also have an indirect signature that penetrates beneath the upper (200 m) layer^20^. The arrival of packets of hurricane-induced near-inertial internal waves at the seabed lags the passage of the hurricane itself by 10–12 days^21^.

Tidal flow interaction with topography is one of the major sources of internal wave energy to the deep ocean. This process can have an immediate effect on flow speed and enhance turbulence near steep obstacles^22,23^ with implications for diapycnal mixing. Globally, the number of topographic features taller than 100 m, based on satellite-derived bathymetry, is estimated at 25 million^24^. Recent instrumental observations have identified high energy dissipation in strongly-sheared layers in descending gravity currents, not directly *over* rough topography but within a few kilometres downstream of *sills*^22,25^, *inside* extended channels^26,27^ or convex-shaped *canyons* incising continental slopes^28^, or near a deep *saddle*^29^.

In this article we quantify the links between *remotely* wind-induced sea-surface current patterns, tidal interaction with topography and deep ocean flow enhancement in order to predict their impact on sediment plume dynamics in the eastern German nodule licence area in the south-eastern segment of the CCZ (Fig. 1a–d). This has implications for the rest of the CCZ mining zone, spanning an area of 6 million km^2^.

## Results

### *In-situ* and remotely-sensed observations

used for this study include a two-year time series of water velocity at 15 to 20 m above the seabed, from three acoustic current meters^30^, as well as sea-surface dynamic topography and surface geostrophic currents derived from satellite altimetry^31^. The height of the bottom Ekman layer *H*_*e*_ did not exceed 16 m. Following Armi and Millard^32^ *H*_*e*_ = 0.4∙(U_*_/*f)*, where *f* = 3∙10^−5^ s^−1^ is the Coriolis parameter, the long-term average velocity *U* = 3.8 cm∙s^−1^ (at mooring 1) and the friction velocity U_*_ over a smooth (flat) seabed is defined as U_*_ = *U*/30. At this level, low frequency velocity approaches the geostrophic balance of the interior, therefore Ekman veering did not contaminate the velocity measurements at 15–20 mab, used as boundary forcing for the high-resolution, non-hydrostatic, hydrodynamic modelling (MIT-gcm^33^).

**Figure 2:**
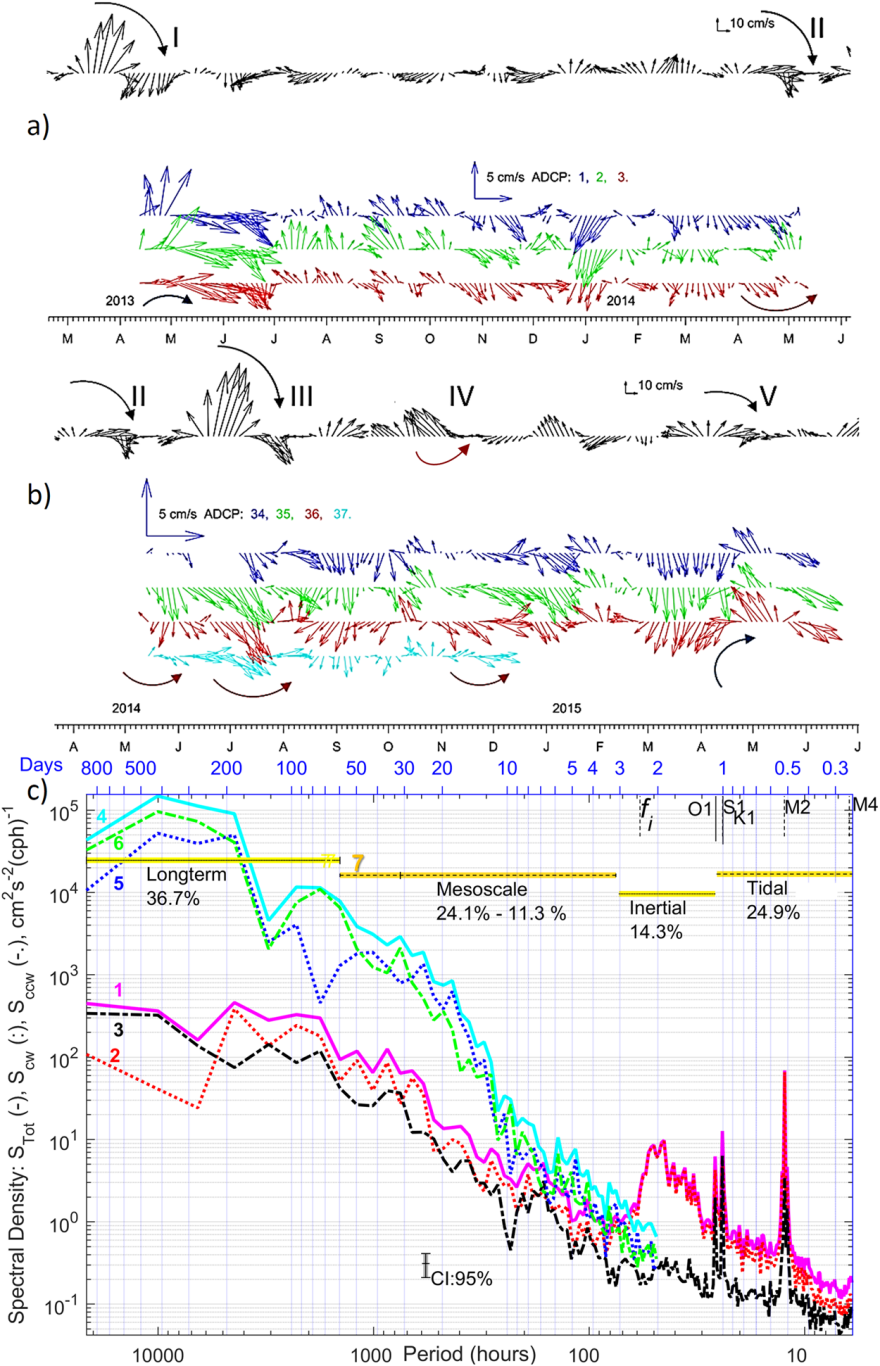
(**a**,**b**) Surface geostrophic velocity (AVISO^31^) (black) and residual currents at mooring N^o^ 1,2,3 (**a**), 34–36 (at the same sites) and 37 (**b**) shown in colours (3-daily averaged, layer 15–20 mab) for two deployment phases. The curved arrows indicate current veering during passage of eddies I-V. (**c**), Rotary spectral density estimates of seabed currents at mooring site 2 are shown with lines ,,**3** for total, clockwise (cw) and counter-clockwise (ccw) components and similar lines ,, for the surface currents. Lomb-Scargle^62,63^ rotary spectra were calculated with unevenly sampled (1 and ¾ hours) data over the period 2013.04.11–2015.06.02. Confidence Intervals (CI = 95%) are included. Integration intervals over long-term, mesoscale, inertial, and tidal + high frequency internal wave’s bands and their contribution (%) in total spectra are shown with lines **7**. High_Res_Figure_2 (https://figshare.com/s/78137cca3607d1266702). Figure was plotted using MATLAB R2015b (http://www.mathworks.com/).

In spring 2013, a consistent increase and doubling of mean flow speed up to 8 cm∙s^−1^ was detected at all three mooring sites over a timespan of several weeks, with peak values (17–24 cm∙s^−1^) exceeding the long-term background average by 4–6 times at 18 mab (Fig. 2a,b). The tidal contribution to total current variability over the whole deployment period is estimated by subtracting the variance of a *synthetic* time series, constructed using discrete harmonic tidal analysis^34^, from the total variance of the *observed* series. The 67 tidal constituents detected, 45 of which with signal to noise ratio above 1, contribute 34.3 ± 0.5% to the total variance. Half of this variance is produced by the largest diurnal and semidiurnal constituents. To identify the sources of variance, capable of providing the other two-thirds of the abyssal energy, we performed rotary spectral, wavelet transform and coherence analyses. This allowed differentiation between the relative contributions of the tides, the near-inertial oscillations often associated with wind and geostrophic shear flow, and the flow induced by mesoscale eddies.

The total spectral density was integrated over super-inertial frequencies (equivalent periods below O_1_ tides 25.82 hrs), near-inertial (25.82–72.54 hrs), and sub-inertial (>72.54 hrs), which include annual, seasonal and mesoscale cycles (horizontal bars on Fig. 2c). Energy fluctuations near seabed in the super-inertial frequency range are driven (variance of 24.9%) by short-frequency oscillations, which include contributions of eight major tidal harmonics (15.8 ± 1.9%), the internal waves and the internal tide, that are not phase-locked, and presumably background noise. Near-inertial oscillations provide 14.3% of spectral energy. Contributions of mesoscale fluctuations vary between 11.3% and 24.1%, for integration limits of 72.54 hrs to 30 days, and 72.54 hrs to 60 days, respectively; equivalent to typical times of mesoscale eddy propagation over the mooring site (Supplementary Info Media 1). The remaining 36.7% of total spectral energy is supplied by lower-frequency seasonal and annual fluctuations.

Combined total and rotary spectra of the daily mean velocities (Fig. 2c) reveal that energy at the sea surface was higher than at the seabed below the inertial frequency *f* (with periods longer than 2.5 days). Clockwise (negative) rotary power density S_cw_ was also higher than counter-clockwise S_ccw_ in both time series at the same frequencies. S_cw_ of the seabed oscillations at near-inertial frequencies was as high as the major diurnal (S_1_, O_1_ and K1) and only slightly lower than semidiurnal (M_2_) tides. Long-term variations total energy also have peak periods of 10, 30, 90 days at the sea surface, and 8, 12, 36, 90 days in the deep ocean layer.

The westward propagation of large surface eddies over the mooring sites is revealed by rotation of velocity vectors near the bed. With a delay following the passage of the eddy centre over the site, currents veered anticyclonically in all three current meter records (Fig. 2a). To investigate the link between the surface and near-bed currents we calculate complex coherence of rotary-decomposed vectors from pairs of spatially-separated time series^35,36^. Complex vector rotary coherence identifies the frequency-dependent relationship between co- and counter-rotation patterns in wind and ocean current fields derived from two time series. We calculate two-sided inner (co-rotation) and outer (counter-rotation) coherences between rotary spectra of two vector time series: surface geostrophic currents derived from satellite altimetry and residual daily-averaged currents measured in the near-seabed layer. If rotary coherence is low (close to zero) then the relationship between two time series in that frequency band is negligible. If coherence is high (near one), and is statistically significant, then two similarly-rotating time series are highly related in that frequency band and their relative phase angle can be determined. To remove the high frequency signal, we de-tided the near-seabed velocity and obtained daily-averaged mean residual current time series (783 days) to compare with the altimetry-derived daily-averaged surface geostrophic velocities at the nearest location, provided by AVISO^31^. A cosine-taper method was applied for spectral averaging. Rotary coherences are shown with high frequency resolution (1/783 cycle per day) (Supplementary Fig. S1a) and their confidence intervals were calculated with the known number of degrees of freedom (9) of the given times-series^37^.

Clockwise co-rotating velocity vectors were coherent above a 95% confidence level for a range of periods with broad maxima at 18, 12 and 8 day periods (Supplementary Fig. S1a). The inner phase angle varies little around −70°, + 90 and + 95° respectively at the same periods (Supplementary Fig. S1b). Counter-rotating vectors exhibited little coherence in nearly all ranges, except for a peak at −18 days in the negative frequency band and at 20, 30 days in a positive band (Supplementary Fig. S1a). The outer phase also was stable around 120° at negative (−18 days), and 90° at positive (30 days) frequency bands (Supplementary Fig. S1b). Weaker coherence between co- and counter-rotating surface and seabed currents above the 95% significance level was also detected at the other two mooring sites at frequencies equivalent to mesoscale processes (1–3 week periods). In all cases, the actual energy contained in the coherent frequency range (Fig. 2c) is low, in particular in the altimeter data which poorly resolves these frequencies. Direct confirmation of the connection between seabed currents and individual sea-surface eddies is not possible without current measurements through the water column. Detected near bed signals were possibly generated by either the surface eddy itself (vertically stretched to the seabed) or its satellite (supplementary) deep vortices^38^. However a wavelet (Morlet) power spectrum of the seabed daily-averaged residual current speed isolates individual peaks in the frequency-time domain (Supplementary Fig. S1c). Wavelet power at each period was normalized by the global wavelet spectrum^39^ and contours of variances above 1 were detected in the mesoscale band (10–30 day periods). Three of these maxima (significance > 95%) occur during the passage of eddies II, III, and IV over the mooring site.

### Mesoscale eddy transit over mooring sites

The “D”-shape of a velocity vector hodograph is a characteristic signature of the passage of full-depth eddies over a mooring current meter^40^ and we detect such signatures in current vectors of the deepest layer. In two of the five cases (I,V) the direction of bottom vector rotation matches that of the surface (Fig. 2), which indicates that associated eddy centres propagated south of the moorings, and that the northern edge of the eddy passed over the moorings. The possibility that eddy centres pass to either north or south of the mooring site explains the presence of the peaks both in the inner coherence (co-rotation) and outer-coherence (counter-rotation) spectra (Supplementary Fig. S1a,b). The eddy-induced low-frequency signal is seen at 4100 m between one and three weeks after the surface signature passes the same location, giving a downward effective transfer rate of 200–600 m∙day^−1^. The daily average horizontal displacement speed of the first eddy centre varied from 5 to 20 cm·s^−1^, and, by inspection, the by time the eddy signal was detected near the seafloor, its surface manifestation had propagated ~50 km westward. Therefore, the eddy’s rotational axis was effectively tilted from the vertical by 4.5^0^ to the west, similar to the tilt of baroclinic eddies formed in the shallower waters (2000 m) of the South China Sea^10^ and on the shelf (200 m) in the Salish Sea^41^. This small axis tilt explains the time delay between seabed and surface eddy signatures.

Several episodes of intensified seabed Eddy Kinetic Energy (EKE) are apparent when no isolated large mesoscale eddy passed directly over mooring sites (spikes on Fig. 1c). However, the sea-surface height (SSH) and geostrophic current maps (Supplement Media 1) indicate the presence of curvature in both SSH and surface velocity fields at these times; corresponding to cyclonic (counter-clockwise) flow rotation in August, October, November 2013 and anticyclonic (clockwise) rotation in February-March 2014. Such eddy-like structures were detected either south or north of the mooring site at distances marginally exceeding the local Rossby radius of deformation. On these occasions the mooring site is evidently affected by weaker eddy peripheries, which result in enhanced currents near the seabed and hence elevated EKE.

### Eddy origin and energy source

Satellite-derived sea-surface dynamic topography^31^ shows that the number of eddies passing over the moorings increased in the second year of observations (eddies II-V on Figs 1b,2b). Such eddies draw their energy mainly from the Central American mountain Gaps Winds, driven by pressure gradient variations between the Pacific and the Gulf of Mexico, and are associated either with North American cold-air outbreaks^42^ or with intrinsic variability of the Trade Winds. Annually 3.5 ± 1.2 Gulf of Tehuantepec (T) and 2.2 ± 0.9 Papagayo (P) large, isolated anticyclonic eddies separate from their origin sites^43,44^ and propagate westward for a year or two. Their initial energy is ultimately exhausted by sub-mesoscale motion^10^, by merging with another eddy, or by collapsing and disintegrating as a result of collision with comparable horizontal-scale features in seabed topography, such as the East Pacific Rise, through quadratic bottom drag and lee wave generation. We tracked all five anticyclonic eddies back to their origin (Supplementary Media 1). The travel duration from the starting point (P, Ia) and (T, Ib-V) to the moorings in the eastern German licence area was 246 ± 64 days, with an average propagation speed of 14 cm·s^−1^. A week before each P and T eddy was formed, a strong wind event (cyclonic tropical storm or hurricane) with wind speed exceeding 15–20 m·s^−1^ was recorded near the eddy origin (Supplementary Table S1, Fig. S2).

**Figure 3:**
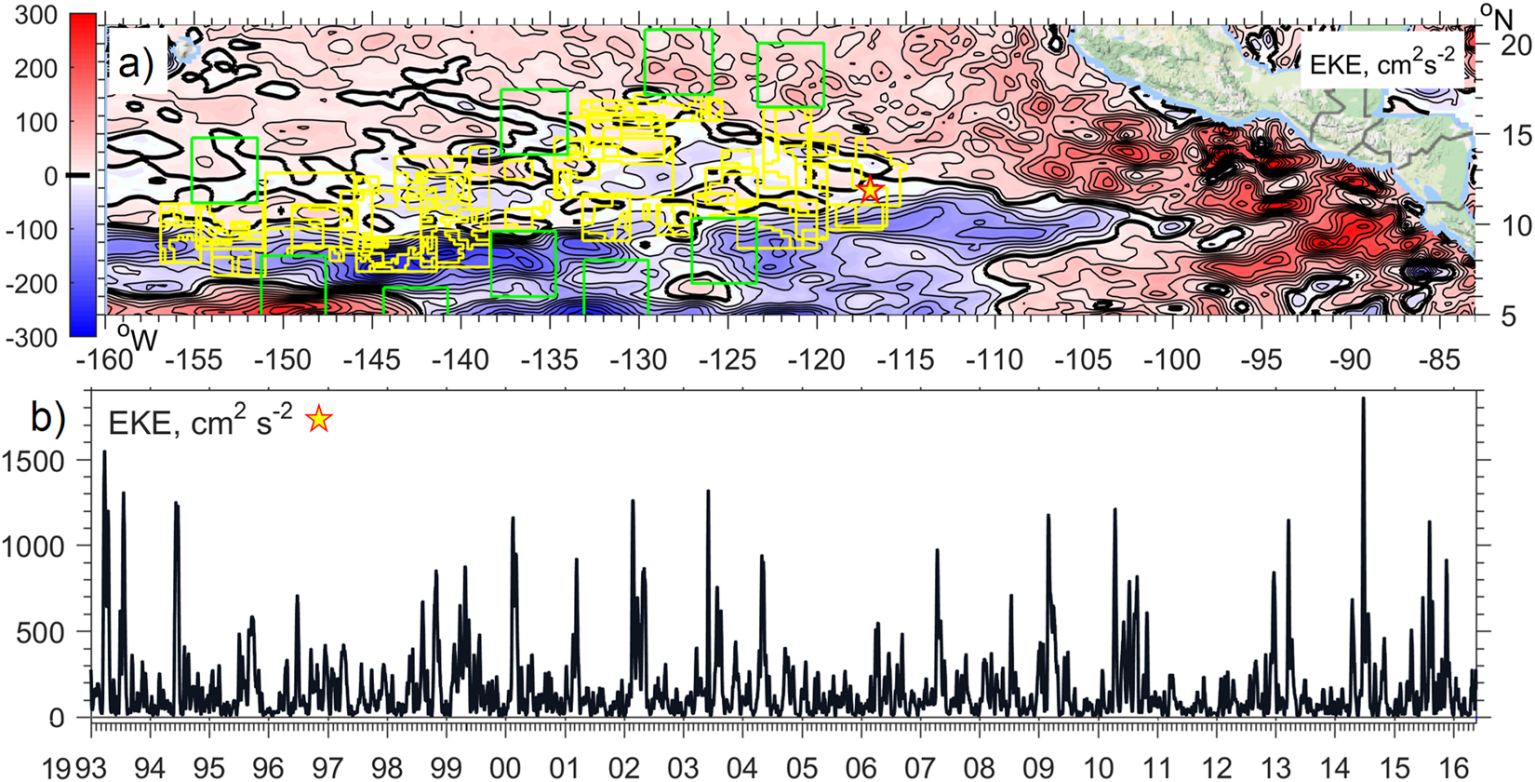
(**a**) The difference between the mean Eddy Kinetic Energy (EKE, defined as half of the horizontal velocity deviation from mean squared, isoline increment is 25 cm^2^∙s^−2^) of the altimetry–derived (AVISO^31^) ocean surface currents between the averaged eight El-Niño and seven La-Niña periods, calculated over 1993–2016. CCZ licence areas in the Tropical Pacific are shown with yellow and protected areas (APEIs) with green lines. (**b**) Surface EKE over the BGR mooring site (star on a map). High_Res_Figure_3 (https://figshare.com/s/2a1da19c8d6afe765e52). Figure was plotted using MATLAB R2015b (http://www.mathworks.com/).The map in this figure was queried from Google Static Map APIs (http://code.google.com/apis/maps/) using the Get_google_map mapping package version 1.4 (https://uk.mathworks.com/matlabcentral/fileexchange/24113-get-google-map).

Potential generation of anticyclonic eddies during the low wind summer seasons and their observed enhanced frequency of occurrence shortly before and during El-Niño periods has been demonstrated in the literature^45^ to be linked with equatorially-generated Kelvin waves, transformed into coastally-trapped waves (CTWs) on their arrival at the Central American coast^45^. The stronger northerlies (offshore Gap Winds) become statistically significant only in May and September of El-Niño years^46^. Baroclinic Kelvin waves with a 30–90 day period are produced in the western and central Pacific under equatorial winds and have an enhanced signal during the Southern Oscillation (SO) warm phase^47^. For the area between 5°N, 20°N, the American coast and 160°W we averaged the surface EKE over the most recent eight low and seven high episodes when the SO Index (6-month running-averaged) was outside a 0.5 standard deviation threshold of the 1993–2016 mean. Across a wide band (600–1000 miles) along the Central American western coast the surface EKE was higher (up to 300 cm^2^·s^−2^) during El-Niño phases in comparison with La-Niña (Fig. 3a). Therefore, and in agreement with^43^, the role of the Southern Oscillation in the modulation of the intensity and increasing the number of eddies at their origin is evident. Taking onto account their slow westward propagation, the spikes in surface EKE above the mooring sites (Fig. 1b,3b) reflect the increase in the number of mesoscale eddies generated prior to and during the latest El-Niño period (mid 2014 - early 2016).

### Modelling Plume Dynamics - the role of internal waves

In order to identify the relative contributions of mesoscale eddy-induced signals and locally-generated internal waves (by tide and mean flow) to the enhancement of abyssal currents, with implications for mining-related sediment plume dynamics, a set of numerical modelling experiments were performed. Internal wave activity in the area is seen in the full-depth repeat profiles of temperature and salinity obtained in the eastern German license area in June 2015. The fine temperature-salinity structure below 3 km depth remained very similar during two days of continuous tow-yo measurement, not being destroyed but vertically displaced by 10–15 m. In this location, the thick, quasi-homogeneous Bottom Boundary Layer (BBL) is composed of Lower Circumpolar Water and extends up to 300 m above the seafloor, with a narrow range of potential temperature θ_*4*_ = 1.465–1.483 °C, salinity *S* = 34.670–34.682 and a very low buoyancy frequency *N* = 0.24 cph (6.7·10^−5^ s^−1^). Bathymetry near the mooring sites reveals a relatively flat landscape, with irregularities of the order of several hundred meters in the vertical and a few kilometres in the horizontal directions distributed, on average, one per 10 km (Supplementary Fig. S3). Ripples in the non-hydrostatic pressure potential field normalised by density (P_NH_∙ρ^−1^) diagnose the spreading of locally generated internal waves (Supplementary Fig. S4a and Media 2). The model-calculated internal wave field was generally weak except at the steep slopes of scattered hills, where the slope angle is close to or exceeds the internal tide propagation angle, the critical value for increasing tidal energy conversion into turbulence. The kinetic energy density of model currents shows a six-fold increase (to more than 1,850 J·m^−2^) near the hills scattered over the model domain (45 × 45 km) in comparison to the averaged background values (335 J·m^−2^) over the relatively flat sea-floor (Supplementary Fig. S5b). These topographic slopes are steep enough to radiate internal tidal energy along beams reflected from seabed at angles 10–15° (upward and downward, Supplementary Fig. S5a). In the horizontal plane, the leading edge of the internal waves radiates slowly from the slopes and over relatively short distances (in the order of several kilometres), thus hardly reaching the nearest mooring site 1, which is located more than 15 km away. Vertical diffusivity averaged over a month in the deepest model layers shows locations of mixing *hotspots* associated with topographic slopes (Supplementary Fig. S4b), affecting dilution rates and spreading of both dissolved and particulate matter plumes.

**Figure 4:**
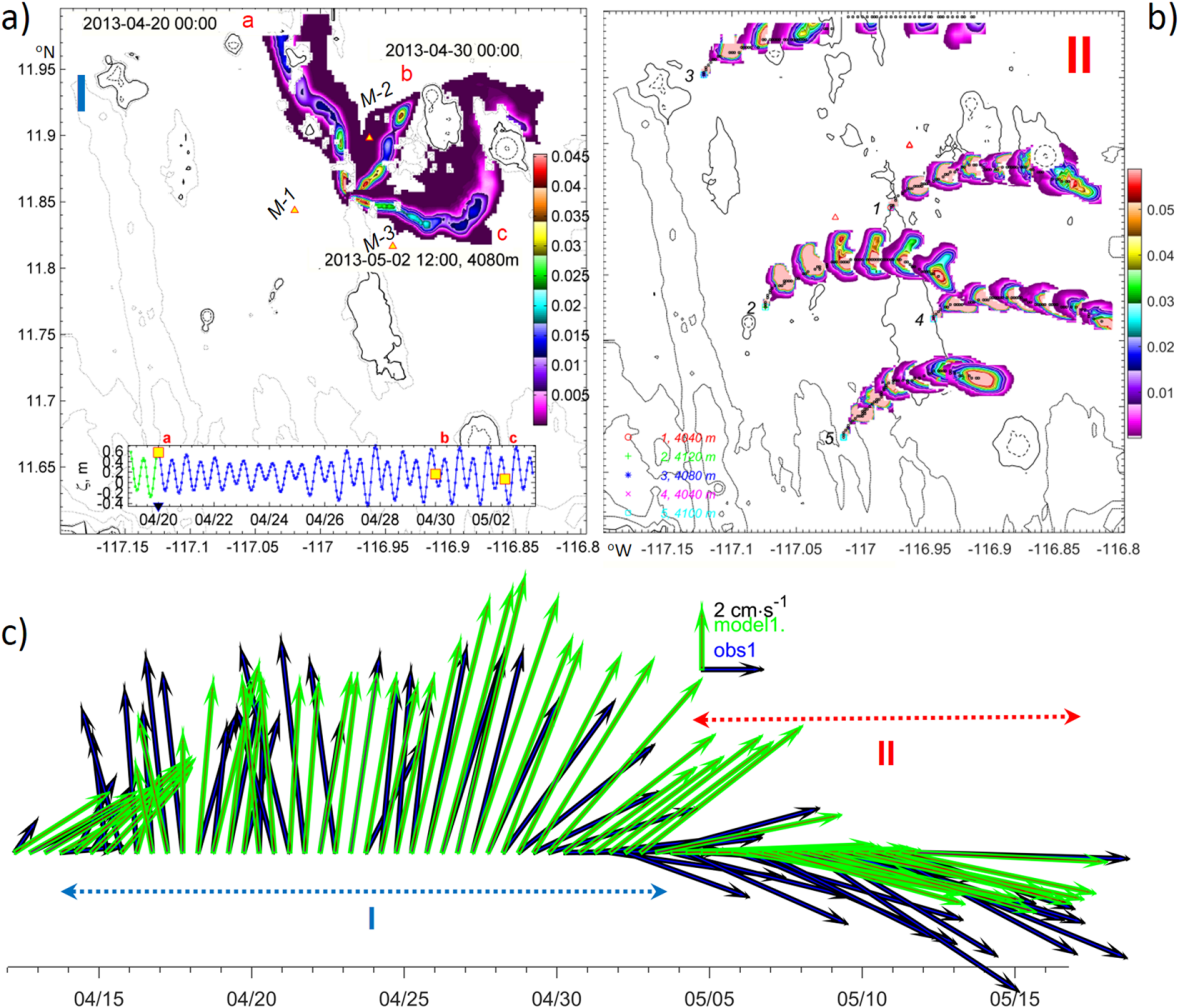
(**a**) Dissolved matter plume core tracks (colours, concentration in parts of unit), formed by neutral tracers released in period  (April 2013) at three different dates (red ,,) marked on inset surface elevation (ζ, m) graph. Mooring sites are labelled with M-1, M-2 and M-3. (**b**) Plume core tracks formed by neutral tracers released in period  from ‘calm’ sources (1,4,5) and two more energetic sites (2,3) adjacent to ‘hotspots’ (shown in 12-hour intervals). (**c**) Observed () and modelled (**)** residual current vectors averaged over 12 hours at 20 mab at mooring site 1. High_Res_Fig. 4 (https://figshare.com/s/1cc75280276f0ead1508). Figure was plotted using MATLAB R2015b (http://www.mathworks.com/).

Dissolved tracer plume modelling is now considered in the context of existing prototype mining scenarios assuming potential leaks of fluids from: (a) the pipes connecting a mining device on the seabed to a floating platform on sea-surface; (b) the mining vehicle (nodule collector) it self; and (c) spreading of the liquid fraction of re-suspended sediments during collection and de-watering processes. A neutrally buoyant tracer plume model was configured in a standard manner^48^. In numerical Experiment I a plume of a dissolved tracer was continuously pumped (at a rate of 1 unit·s^−1^) from the near-bottom model cell in the centre of the mooring triangle. The resulting spreading aligns with the eddy-induced residual flow in April 2013 (3 sequential horizontal snapshots, Fig. 4a). The model indicates that the near-bottom turbulence can vary significantly over short distances (3–4 km) forming *hotspots* on the lee-side of topographic obstacles (Supplementary Fig. S4b). Therefore, in Experiment II (Fig. 4b) five spatially distinct tracer injections, with the same discharge rate, were made into the lowest model layers for one hour in the period 2^nd^–5^th^ May 2013, i.e. after the eddy-induced veering signal had vanished and the observed steady eastward mean flow was re-established. We traced the evolution of the plumes released from sites 1, 4, 5 (far from *hotspots*) and 2,3 (near *hotspots*) (Supplementary Fig. S4a,b indicates tracer release locations). Near the topographically-enhanced mixing zones and in the downstream flow, a higher plume core dilution rate was obtained. Maximum dissolved tracer concentrations reduce significantly immediately after plumes 2 and 3 pass through mixing *hotspots*, while in plumes 1,4,5 travelling over calm and less energetic sites, concentrations remain higher for a longer period of time (colour lines in supplementary Fig. S6a). Dilution was sufficient to disperse material up to 120 m into the mixed BBL within only a day (Supplementary Fig. S6 panels 1–5). Both Experiments I and II reveal that tracer dispersion is controlled by vertical diapycnal mixing in combination with horizontally and vertically sheared currents.

**Figure 5:**
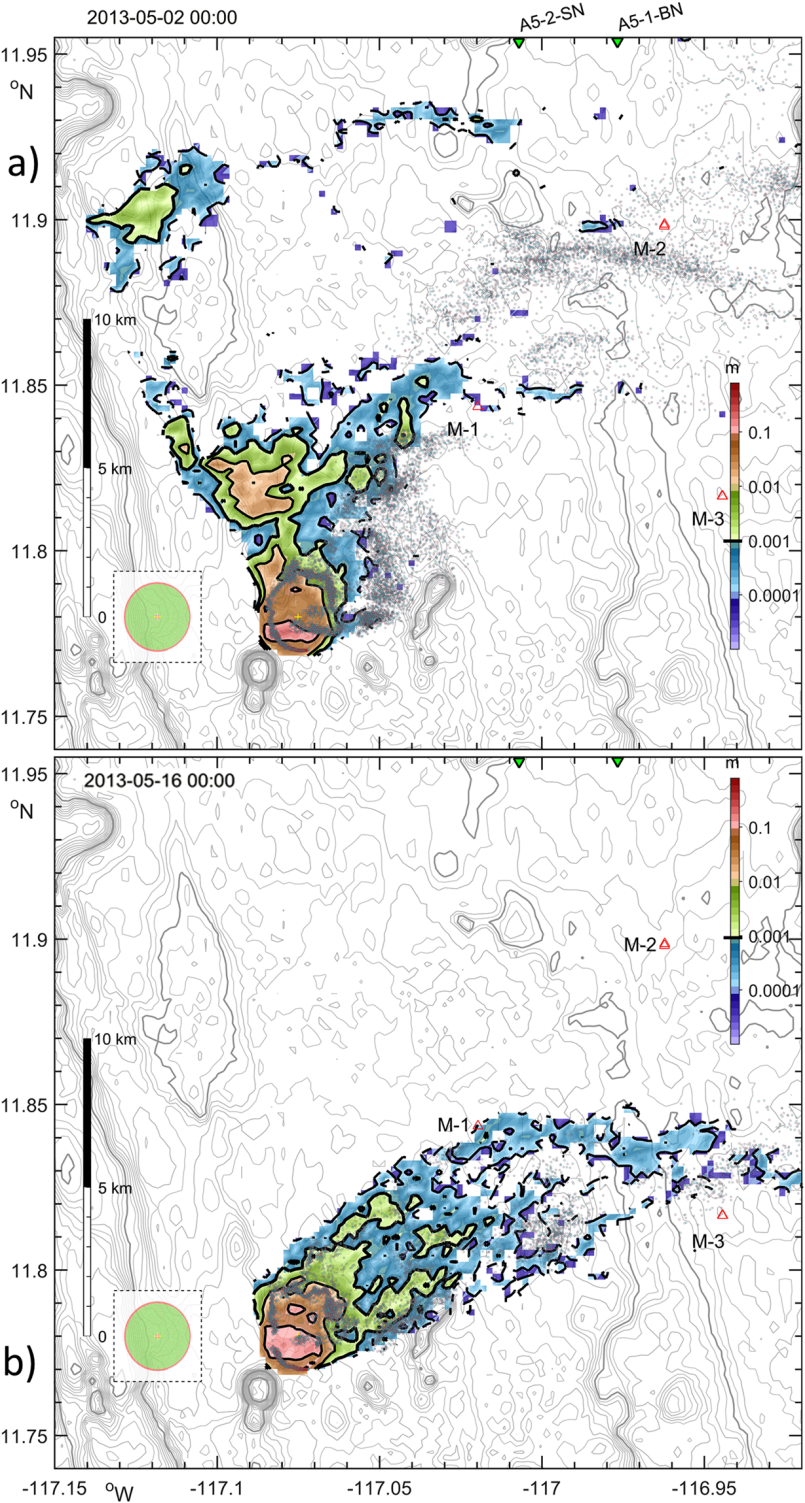
(**a**) Model particulate plume on the 10^th^ day after start release (at the end of eddy  passing period) containing 5.7·10^5^ individual particles suspended in a water (grey dots) and settled on seafloor (colours). The area with substantial accumulated sediment layer thickness (in m) is shown over bathymetry (grey lines, 50 m). Points along the nodule collector tracks were aligned with equally-spaced Archimedes spiral, and shown on a dashed in-cut to indicate the scale of the harvested zone during the last day (red) and since the beginning of experiment (green). Red and green triangles indicate three mooring sites, labelled with M-1, M-2 and M-3, and two sediment core sampling sites^53^ respectively. (**b**), Settling footprint after 10 days of a similar SPM release over period  without eddy impact. High_Res_Fig. 5 (https://figshare.com/s/5472457ce071bd654305) and animations are available on-line via *Supplementary Info* Media3_2D_plume_movie.gif (https://figshare.com/s/abd96d10a22263c42e3b). Figure and media animation were plotted using MATLAB R2015b (http://www.mathworks.com/).

Particle-based plume modelling. A mining-related SPM plume is expected to contain natural sediments from the top 10–15 cm of the ocean floor and nodule fragments deriving from extraction and crushing techniques. Fine sediments that are dispersed close to the seafloor, potentially over vast areas, will affect deep-sea ecosystem structure and functioning through the burial of meiobenthos, clogging of the respiratory surfaces of filter feeders and through coverage and dilution the already impoverished food supply. Very little is known about the potential eco-toxicological effects of crushed nodules by comparison with exploration of massive sulphides near hydrothermal vents systems^49^, but draping of fine sediments could further impact deep-sea species abundance and diversity. The goal of Experiment III was to define boundaries of the area affected by the spreading of a particulate sediment plume. Numerical simulation was based on a conceptual mining scenario (see Methods); a plume consisting of half a million suspended particles, released at 5 mab, was traced for several weeks (Fig. 5a,b) over two contrasting periods with (a) and without (b) eddy-induced impact. Within 10 days, more than half of all particles had settled within several km of the ‘harvesting’ vehicle; the rest remained suspended or had exited the model boundary. Both the shape of the SPM plume and its sedimentation footprint on the seafloor closely mimic the background circulation pattern, associated in Experiment III with current veering induced by the eddy passage (a), and closer to Gaussian distribution under steadier and weaker eastward flow (b) afterwards. The settled layer thickness was higher near the source and outside mixing *hotspots*. After 10 days the area over which the re-deposited sediment thickness exceeded 5 cm did not extend further than 1.25 km from the mining zone. The isolines 1 and 0.1 cm were found maximum at respectively ~5 and ~12 km away from the source and in the direction of dominant high eddy-induced flow (Fig. 5a), and substantially closer (1.5 and 6 km respectively) in period II (Fig. 5b). These estimates are not dissimilar to previous observations^50^.

**Figure 6:**
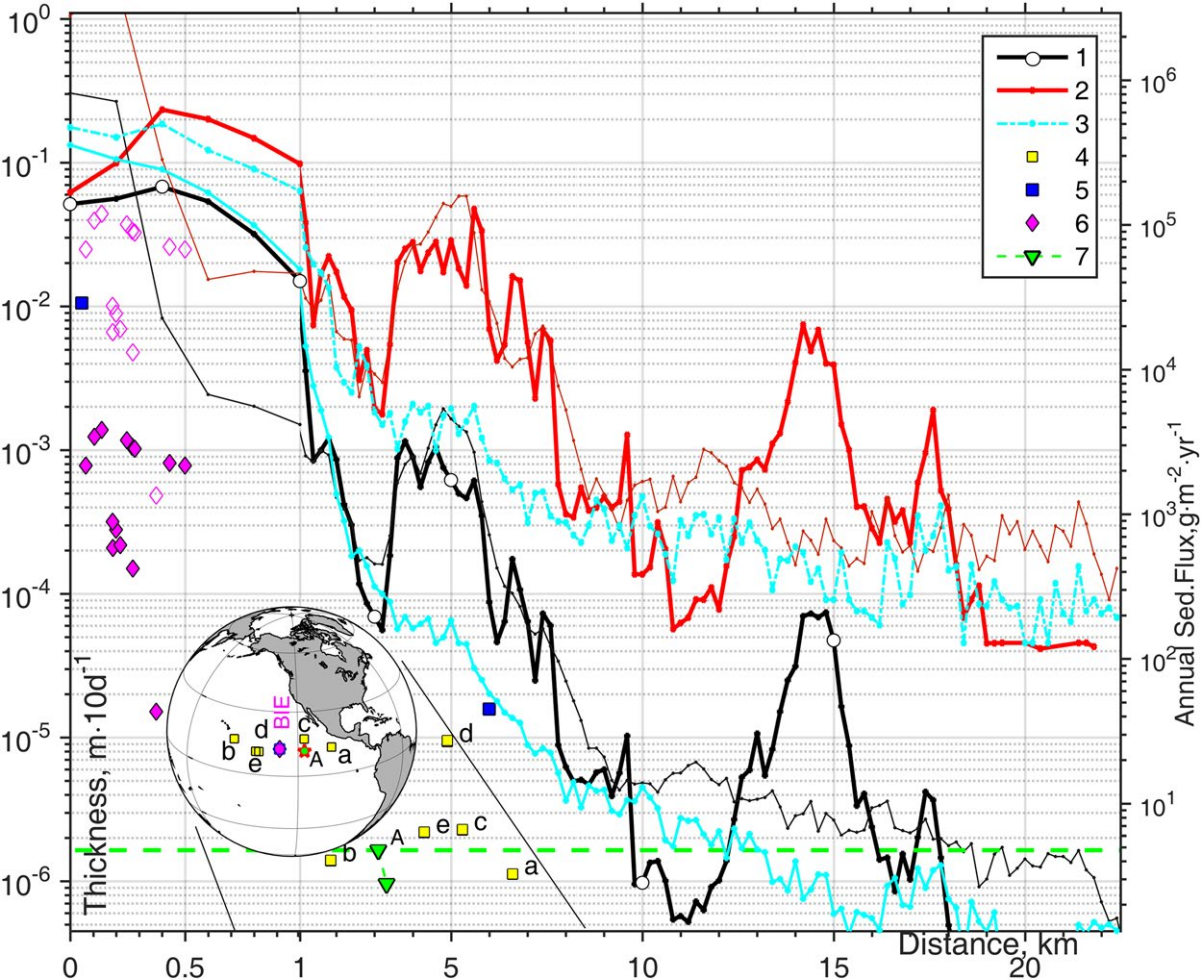
Settled sediment thickness (m) as a function of distance from the source are shown with ***1*** black (averaged) and  red (maximum) lines computed in numerical Experiments III under eddy impact using two vertical mixing schemes: KL10^68^ (bold) and PP81^67^ (thin). Cyan lines  show the averaged (solid) and maximum (dashed) thickness of sediments settled during the other 10 days of SPM release, when the impact of the eddy vanished. Yellow symbols ***4*** show JGOFS^77–80^ sediment trap rates and their location in Eastern Pacific. Symbols , show visual and measured data from 15 sediment traps collected in BIE^50,51^, while empty diamonds show BIE trap values scaled by suspended mass ratio R = 31.8 over 10 days between this numerical experiment (454,756*t*) and *in-situ* BIE trials (1,427*t* of sediments were dispersed during 19 days at a rate of 4.2 kg·s^−1^ by a six-meter-wide “benthic disturber” that was towed in 49 parallel rows within a 3300 m × 150 m polygon)^51^. Green line ***7*** indicates the natural sedimentation rate at two *A5* stations^53^. High_Res_Fig. 6 (https://figshare.com/s/d984083a59832f4227ea) and Table 3 are available on-line via *Supplementary Info*. Figure was plotted using MATLAB R2015b (http://www.mathworks.com). The map in this figure was generated by MATLAB R2015b with M_Map (a mapping package, http://www.eos.ubc.ca/~rich/map.html).

Scalable comparison and model validation is possible using data obtained from the centre of the CCZ claims area in a similar environments in a joint NOAA/Russian Benthic Impact Experiment (BIE)^50^. Post-impact observations and analyses of 15 sediment traps^51^ revealed that the mean distribution of sediments collected in traps increased over 2 orders of magnitude (0.03–1 mm) and was proportional to the distance from the source. The maximum was aligned with the mean flow direction. Immediately near the tow-impacted zone, nodules were buried under 2 cm of fresh sediments. This is similar to the blanketing thickness (5 cm) at the same distance from the source calculated in our model of the eastern CCZ German licence area. Applying a scaling ratio between sediments dispersed in our numerical experiment and in BIE (R = 31.8) to the observed sediment traps content also shows similar (1–4 cm) scaled thickness for near-field results (Fig. 6 and Supplementary Info Table 3). The natural level of background sedimentation in the Central Pacific, accumulated during one thousand years (1–6 mm)^52,53^, is reached within just 10 days under the mining scenario simulated here (Fig. 6, Supplementary Info Table 3). The re-deposition of plume SPM at this scale is expected to have a huge impact^5^ on the generally non-resilient deep ocean ecosystem, which could be prone to irreversible changes under such enormous pressure.

### Implications

In the eastern part of the Central Tropical Pacific/CCZ, at an average water depth of ~4100 m, tidal energy contributes only one third to abyssal current variability over smooth topography. We show that the other notable energy controlling factors are (i) *near-inertial* oscillations, induced by wind and geostrophic shear, which are nearly as energetic as (ii) *tidal* contributions, and (iii) mesoscale ocean *eddies*, generated 3500 km away under the influence of the Central American Gap Winds. The remotely-generated eddies occasionally dominate the near-bed current regime, and, for a duration of several weeks, can induce a five-fold increase in mean flow speed, with maximum values above published thresholds for anticipated resuspension of fine-grained deep-sea muds^54^. This implies that resuspension of deep-sea sediments might be a regular, natural process. We have demonstrated that during the passage of large isolated eddies the abyssal velocity signal is significantly coherent with the surface current field, and we model its influence on plume advection reflecting the dominant seabed flow. Numerical model experiments indicate that even in a weakly-stratified, thick (300 m) BBL the presence of topographically induced oscillatory motions, such as internal waves with a higher than tidal frequency, lead to formation of turbulent mixing *hotspots*, where (a) dilution rate in the dissolved plume core is 3–5 times higher than background values at short (few km) distance from steep slopes (Supplementary Fig. S6a), and (b) lead to substantial variations in the site-specific horizontal spreading plume pattern and re-deposited thickness of the re-suspended sediments (Fig. 5a,b).

Strong winds blowing for several days through narrow gaps in high mountains are capable of inducing mesoscale eddy shedding from coastal current jets, some 3500 km distant. The ‘memory’ of such remote winds is preserved in self-propagating mesoscale eddies for year(s) and *surprisingly* they can have a significant impact on steering near-seabed waters in the abyssal ocean thousands of kilometres from the coastline. Detecting and tracing eddies *in advance* using the methodology we show here could help anticipate and assess the behaviour of both natural and man-made plumes in a representative range of conditions over potential mining sites, as well as over surrounding protected areas (e.g. APEI and Preservation Reference Zones). For planning mining operations and environmental management schemes, including preservation measures, the prediction of the abyssal footprint of surface eddies is vital, although limited by the spatial (28 km) resolution of currently available satellite products. Substantial improvements in resolution of meso- and sub-mesoscale sea surfaces dynamics is expected with transition from the Profile to Swath altimetry with the launch of the new Surface Water and Ocean Topography (SWOT)^55^ satellite mission in 2020.

Measures to minimize mining plume impacts on abyssal habitat communities could be species-dependent and even dissimilar between life-phases. It is not yet known exactly how increased plume dispersion during high flow periods will influence larval dispersal, while low-flow regimes with lower spreading rates and greater blanketing may adversely affect abundance and diversity. To identify the actual burial tolerance boundary and thresholds associated with SPM burden, alongside eutrophication responses initiated by re-suspension of organic compounds, more observational evidence is required, such as anthropogenic impact studies on shallow water species^56,57^. A recommended approach to prediction and environmental assessment of plume development during mineral harvesting would be to nest a fully-operational short-term forecasting model within eddy-resolving regional/global ocean (e.g. HYCOM^58^) and atmospheric (e.g. GFS^59^) simulations. These models would ideally be able to assimilate sea surface and water column data provided by altimetry, autonomous gliders, Argo floats or similar measuring platforms, and would be accompanied by a sediment-transport model which takes into account the cohesive properties and aggregation of fine-grained sediments.

## Methods

### Field data analysis

The site of the moorings in the German license area of the CCZ (Fig. 1) was chosen to minimise the role of flow interaction with topography by reference to detailed multibeam bathymetry acquired with a SIMRAD EM 120 swath mapping system from R/V Kilo Moana^30,60^. A relatively flat abyssal site was selected, remote from seamounts and guyots (Supplementary Fig. S1). For 13 months from 11^th^ April 2013, vertical profiles of zonal (u), meridional (v) and vertical (w) velocities were obtained with a 1-hourly sampling rate in 10 layers with 1.5-m vertical bin-size and acceptable data in the range of 15–20 metres above the seabed by three RDI-ADCP (WH 600 kHz) upward-looking current meters deployed by BGR during their MANGAN 2013 cruise^30,61^. The moorings (1, 2, 3) were placed 8 km apart in a water depth of around 4120 m at the vertices of an equilateral triangle centred at 11.86°N, 116.97°W (Fig. 4a, Fig. 5). The instruments were redeployed at the same depths and geographic locations for another 13 months on 12^th^ May 2014 (moorings 34, 35, 36) together with the fourth mooring (37; RDI-ADCP WH 150 kHz), all with 45-minute sampling rates. The mean current speed was below 4 cm∙s^−1^ and maximum values (17–24 cm∙s^−1^) were recorded in April-May 2013. The MATLAB T_TIDE package^34^ was used for harmonic tidal analysis and Lomb^62^ -Scargle^63^ rotary spectra were calculated with unevenly sampled (1 and ¾ hours) current time series. Full-depth Conductivity-Temperature-Depth (CTD) profiles were obtained a short (few km) distance from the moorings during their deployment and recovery and were used to initialise stratification and provide lateral boundary conditions to the hydrodynamic model. Vertical CTD profiling in a tow-yo mode was performed on station 93 in June 2015 during the SO240 cruise with R/V Sonne^61^ and indicate persistence of weak stratification in a thick (>300 m) BBL. Stratification slightly increased at the upper margin of the BBL (3600–3700 m) over a two year period (2013–2015). During the same period, temperature increased by 0.004 °C (Supplementary Fig. 5c), whereas salinity decreased by 0.008 near the seabed. As stratification and the geometry of topographic features are key factors governing internal wave generation, we used high-resolution bathymetry^60^ for the model.

### Eddy back-tracking algorithm

Eddy back-tracking was based on detection of its centre defined as the maximum sea surface height value within a 200–300 km search radius in a sequence of daily digital maps^31^. For example, eddy I was created from merging Papagayo (Ia) and Tehuantepec (Ib) eddies near 99°W on the 25^th^ October 2012, each associated with its own deep atmospheric cyclone in the preceding week. Eddy Ia separated from the Costa Rica Coastal Current (CRCC) near the Guatemalan coast on 18^th^ June 2012, in the presence of hurricane Carlotta which developed to full strength (175 km∙h^−1^) on 14–15^th^ June at 9°N, 90°W^64^. The wind pattern (NCEP GFS^59^) and the surface currents (HYCOM^58^) during this week are shown in Supplementary Fig. S2. Eddy Ib was formed on 11^th^ September 2012 at the northern periphery of the Tehuantepec Gulf soon after the passage of a low atmospheric pressure tropical wave over its southern sector, which later strengthened into Tropical Storm Kristy. A similar sequence of events was detected a week before each of the other four traced eddies were formed during the windy autumn seasons of 2013 and 2014 (Supplementary Table S1, Media 1).

### MIT-gcm model configuration

A high-resolution, non-hydrostatic ocean model was developed to investigate mining plume dynamics in the region and to detect the location of enhanced mixing patches driven by the interaction of tidal and mean flow with bathymetry. We introduced a telescopic increase in horizontal grid spacing up to 1 km at the model’s lateral open boundaries, aiming to reduce the influence of spurious boundary reflection. Grid stretching was performed with a shifted hyperbolic tangent function^65^ and 80% of the model domain has horizontal resolution 200 m (Fig. 4a) and 20 m in vertical. We also added extra terms to the MIT-gcm^33^ momentum balance equations: the tidal potential computed with 8 major tidal harmonics using an inverse tidal solution^66^, and the measured daily-averaged residual currents. A nodal correction and tide origin time were adjusted to exactly match the model tidal phase with the observed one. Model runs duration was restricted to five weeks in spring 2013. The hydrodynamic model was spun-up for 10 days, after which the SPM model was initiated. For the surface boundary condition we used an implicit linear free-surface and for the bottom boundary, no slip bottom conditions were applied. Background horizontal eddy viscosity and diffusivity were set as 0.1 m^2^s^−1^. This estimate is comparable to the values of numerical diffusion for similar fine-scale horizontal grids^48^. Background vertical eddy viscosity was set to 10^−4^ m^2^s^−1^. The presence of locally (topographically) generated nonlinear internal waves could lead to strong velocity shear and mixing. To enable the model to resolve these features we applied the Pacanowski-Philander vertical mixing scheme^67^ with Richardson number dependant parameterisation for turbulent closure of vertical viscosity and diffusivity. Experiments with energy dissipation computed with a KL10 closure scheme^68^ based on sorting of vertical density profile demonstrated similar but more apparent internal waves dynamics (Supplementary Fig S4a and Media 2). The relation between the gradient of the seafloor (*S*_*b*_) and the characteristic path of internal tide propagation (*S*_*w*_ = *[(ω*^2^ − *f*^2^*)/(N*^2^ − *ω*^2^]^*0.5*^, where *ω, f* and *N* are the tidal, Coriolis and buoyancy frequencies) is a key factor determining the nature of tidal energy conversion. In areas with *subcritical* slope, where the ratio *α* = *S*_*w*_/*S*_*b*_ < 1, low mode internal waves were generated in the model. Tidal energy conversion rates increased^69^ in areas with a *supercritical* slope (α ≥ 1) and tidal energy propagation was concentrated in narrow beams. Integrated over time and over depth available potential (*APE*) and kinetic energy fluxes *Ek* = *Ek*_0_ + *Ek*_*bc*_ were computed with decomposition of velocity into barotropic (*U,V*), baroclinic (*u*′,*v*′) and vertical (*w*) components and were defined as *APE* = *ρ*′·*g*′·*z*′, *Ek*_0_ = 0.5*·ρ*_*o*_*·(U*^2^ + *V*^2^) and *Ek*_*bc*_ = *0.5·ρ*_*o*_·(*u*′^2^ + *v*′^2^ + *w*^2^) respectively, where *ρ*_*o*_ denotes the reference background density, *ρ’* reflects density perturbations, *z’* is vertical displacement of isopycnals and *g* is gravity. With low values of Courant number c ≈ 0.0015, the second order non-linear flux limiter advection schemes (such as ‘Superbee’^70^) are preferable^33^ and were applied for experiments with tracer releases.

### Internal tides in the model and observations

The modelled vertical distribution of horizontal currents along the long transect (black A-B line, Supplementary Fig. S4) indicates that enhanced kinetic energy flux concentrated in narrow Internal Tide (IT) beams emanate at a small angle *S*_*w*_ upward and downward from the supercritical slope of the hill at 10 km distance from A (Supplementary Fig. S5a). Isopycnal surfaces are displaced vertically along these beams. In the model the amplitude of displacement fades out quickly with distance from steep slopes. To estimate spatial variations Kinetic Energy was integrated vertically over a layer between the seabed Z_b_ and upper BBL limit Z_T_ and within *x* = 2.5 km segments along the A-B transect ${E}_{k}={\langle 0.5{\int }_{{z}_{b}}^{{z}_{T}}({U}^{2}+{V}^{2})\cdot dz\rangle }_{X}$. Higher values of *E*_*K*_ were detected near steep slopes above the trench and hill (Supplementary Fig. S5b). The internal tide wavelength is *L*_*M2*_ = *h·cotang (S*_*w*_*)*, where *S*_*w*_ ≈ 10° is the beam angle (M_2_ characteristic path) and h = 400 m is the affected layer thickness. The analytical value *L*_*M2*_ = 2.3 km is close to the numerical model results of 2.2–2.7 km for the distance between peaks (troughs) along the isopycnals that are vertically displaced by propagating internal tides. In the model, the vertical amplitude of isopycnal displacement (ξ) varies between 30 and 50 m above the steep topographic slopes and ξ = 20 m elsewhere. These model results are in agreement with the range of displacement amplitudes detected from tow-yo CTD profiles^61^, performed over the 10 km transect C-D during a single tidal cycle in the area (Station 93, Supplementary Fig. S5a,c).

Hydrodynamic model assessment and performance skill estimates were based on regression analysis^71,72^ of the model results against velocity measurements in the deepest ocean layer at all three mooring sites (Supplementary Table S2). Observed and modelled 1-hourly sub-sampled and residual (de-tided 12-hourly averaged) zonal ${\langle u\rangle }_{{d}}$, meridional ${\langle v\rangle }_{{d}}$ velocities were at least in *very good (0.7*2*)* agreement, corresponding to the Willmott^71^ index. The difference between one-hourly sampled modelled and observed currents also does not exceed 28%, which is substantially better than “factor of two agreements” published for other deep sea simulations^73^. The model demonstrates better performance skills at moorings sites 1 and 2, than at site 3 which is topographically-sheltered from northward flow.

### Plume model experiments and validation

The first numerical plume Experiment I was designed to detect the response of spreading *dissolved* matter to a passing eddy. The pumping of neutrally-buoyant tracer into the model layer 20 mab was set to begin 24 hours after model spin-up with a constant rate (1 unit·s^−1^) (Fig. 4a). In Experiment II, neutrally buoyant tracers were injected into the lowest model layers (1 unit·s^−1^) for one hour and were traced during the period 2^nd^ − 5^th^ May 2013, after the eddy-influenced veering of seabed currents had vanished and generally eastward flow was re-established. Tracer injections were performed at 5 different locations, including mixing *hotspots* (N° 2, 3) and relatively calm (N° 1, 4, 5) sites (Fig. 4b). A passive tracer plume model validation strategy is based on scaling of the output fields by the initial concentration constant, obtained from the technical specification of the dispersion source, and should be accompanied with an adequate *in-situ* sampling of chemical compounds. No such observations exists for the deep ocean but in a shelf environment the MIT-gcm plume module used here was found to be in a good agreement with observations obtained by a fluorimeter mounted on a Scanfish following a release of rhodamine fluorescent tracer^48^. On the shelf, the model was capable of replicating with high accuracy the observed dye patch evolution, the enhancement of dye dispersion and its effective horizontal mixing on a horizontal scale that is relevant to this study, 1 to 10 km. The spreading effect of lateral numerical diffusivity also was found to be insignificant.

In Experiment III a suspended particulate matter (SPM) plume was simulated by tracking the transport of 5.7·10^−5^ discrete particles, representative of a realistic nodule mining situation (personal communication Kevin Murphy, Environmental Resources Management). The scenario involves the removal of the upper 15 cm of the sediment layer, of which 90% is expelled and suspended near the seafloor by a single nodule prototype ‘collector’ that mines in a ‘lawn-mowing’ pattern (along a zigzag or spiral track) at a speed 0.3 m·s^−1^. Natural sediment grain size distribution in the range 1.15 to 2·10^3^ μm were based on the analysis of sediment samples recently obtained in a nearby license claim area^74^. The dry weight and bulk sediment densities were assumed 2.65 and 0.8 g·cm^3^ respectively. Sinking and settling speeds were determined via a Reynolds number based approach^75,76^. Every minute (SPM model time step), 20 particles of randomly selected size and mimicking a discharge rate of 278 kg·s^−1^ were suspended within 10 mab layer. During 10 (19) days and working 57% of the time, a single collector would suspend in total 240,000 (454, 756) tonnes of sediments.

The SPM model was found to be robust in a series of sensitivity tests in which horizontal and vertical mixing coefficients were varied by three orders of magnitude. The model produced very similar spreading patterns of suspended particles and its seabed footprint under the given flow regime and only marginally smoothed contours in experiments with higher diffusivity. However, in sensitivity tests with varying ambient flow speed, differences were detected in settled particle size distributions. The SPM horizontal settling distribution after 10 days of nodule harvesting along the non-overlapping collector tracks, aligned with equally-spaced Archimedes spiral, are shown (Fig. 5a,b) for the case with horizontal and vertical mixing coefficients *K*_*x*_ = 0.1 m^2^s^−1^ and *K*_*z*_ derived from the MIT-gcm model runs over two distinguished periods: a) in a presence of eddy-induced enhanced currents and b) afterward.

Sediment traps could potentially be used for validation, providing reference values of Annual Settling Flux (ASF, g·m^−2^·yr^−1^). However, these would have to be used with caution due to uncertainties regarding their effectiveness associated with tilt of the collector surface, height above seabed, turbulence introduced by the trap shape itself and surrounding micro relief, natural re-suspension and the geologically short duration of such installations. Regional measurements data in Pacific^77–80^ are available in a global sedimentation rates database^78^ and are used as proxy for comparison and validation of the SPM model and the averaged settled layer thickness (Fig. 6 and Supplementary Info Table 3). Burial compaction reduces the porosity of bulk sediment (50–90%) and almost linearly increases its dry bulk density over depth in the range 0.2–0.9 g·cm^−3^ in the abyssal Pacific^81^. Applying a constant typical bulk density in the upper sediment layer, here 0.8 g·cm^−3^, could result in ~50% uncertainty of the thickness estimates. Ultimately, any serious validation of model results would involve testing of the mining equipment and synchronous monitoring of plume dispersal and sediment deposition (e.g. using AUV photo imaging).

### Data availability

The altimeter products (seas surface elevation and geostrophic velocity) were provided by AVISO^31^ and Wind data were obtained from NOAA NCEP^59^. The bathymetric and current meter data and the R/V Kilo Moana cruise reports (2013, 2014) are available from the originators [AV] upon request; the CTD cast data and R/V Sonne (SO240) cruise report^61^ are publicly accessible through the GEOMAR OSIS (Ocean Science Information System) portal http://portal.geomar.de/kdmi.

## Electronic supplementary material

Supplementary information

Supplementary Media 1

Supplementary Media 2

Supplementary Media 3

Supplementary Media 4
