# Publisher Correction: Impact of remotely generated eddies on plume dispersion at abyssal mining sites in the Pacific

**DOI:** 10.1038/s41598-018-25181-6

**Published:** 2018-05-04

**Authors:** Dmitry Aleynik, Mark E. Inall, Andrew Dale, Annemiek Vink

**Affiliations:** 1 SAMS, Scottish Association for Marine Science, Scottish Marine Institute, Oban, PA37 1QA UK; 2 BGR, Bundesanstalt für Geowissenschaften und Rohstoffe, Stilleweg 2, 30655 Hannover, Germany; 3 University of Edinburgh, School of Geosciences, Edinburgh, EH9 3FE UK

Correction to: *Scientific Reports* 10.1038/s41598-017-16912-2, published online 05 December 2017

In the original version of this Article, the ORCID ID and an additional affiliation for Mark E. Inall was omitted. The correct affiliations are listed below:

SAMS, Scottish Association for Marine Science, Scottish Marine Institute, Oban, PA37 1QA, UK

Dmitry Aleynik, Mark E. Inall & Andrew Dale

BGR, Bundesanstalt für Geowissenschaften und Rohstoffe, Stilleweg 2, 30655, Hannover, Germany

Annemiek Vink

University of Edinburgh, School of Geosciences, Edinburgh, EH9 3FE, UK

Mark E. Inall

The original version of this Article also contained errors in the Materials and Methods section under subheading ‘MIT-gcm model configuration’.

“A was developed to investigate mining pluhigh-resolution, non-hydrostatic ocean modelme dynamics in the region and to detect the location of enhanced mixing patches driven by the interaction of tidal and mean flow with bathymetry.”.

now reads:

“A high-resolution, non-hydrostatic ocean model was developed to investigate mining plume dynamics in the region and to detect the location of enhanced mixing patches driven by the interaction of tidal and mean flow with bathymetry.”.

The original version of this Article also contained errors in the Results section under subheading ‘Modelling Plume Dynamics - the role of internal waves.’.

“The isolines 1 and 0.1 cm were found at respectively ~5 and ~12 km away from the source and in the direction of dominant high eddy-induced flow (Fig. 5a), and substantially closer (1.5 and 6 km respectively) in period II (Fig. 5b).”

now reads:

“The isolines 1 and 0.1 cm were found maximum at respectively ~5 and ~12 km away from the source and in the direction of dominant high eddy-induced flow (Fig. 5a), and substantially closer (1.5 and 6 km respectively) in period II (Fig. 5b).”

These errors have now been corrected in the HTML and PDF versions of the Article, and in the accompanying Supplemental Material.

