# Supplementary material for: Impact of remotely generated eddies on plume dispersion at abyssal mining sites in the Pacific
Source: Sci Rep. 2017 Dec 5;7:16959. doi: 10.1038/s41598-017-16912-2 (PMC5717004; doi:10.1038/s41598-017-16912-2)
Supplement: Supplementary file 1 — Supplementary information [file 41598_2017_16912_MOESM1_ESM.pdf]

Scientific REPORTS | 7: *16959* | DOI:10.1038/s41598-017-16912-2

Impact of remotely generated eddies on plume dispersion at abyssal mining sites in the Pacific

Dmitry Aleynik[1]*, Mark E. Inall[1,3+], Andrew Dale[1+], Annemiek Vink[2+]

[1] SAMS, Scottish Association for Marine Science, Scottish Marine Institute, Oban, PA37 1QA, UK
[2] BGR, Bundesanstalt für Geowissenschaften und Rohstoffe, Stilleweg 2, 30655 Hannover, Germany
[3] University of Edinburgh, School of Geosciences, Edinburgh, EH9 3FE, UK

[*] dmitry.aleynik@sams.ac.uk
[+] These authors contributed equally to this work

Supplementary Media 1 | Animation of the Sea Surface Height (a) and Geostrophic Velocity (b) charts derived from AVISO[31] shown in 10 day intervals over CCZ licence area (white contour) and BGR moorings locations shown with pentagram. Mesoscale eddy numbers (I-V) refer to the article text. Media1_Eddies_tracking_SSH_V.gif 17Mb (https://figshare.com/s/5b3df66f9dc2a657f830) Animation was generated using MATLAB R2015b (http://www.mathworks.com/).The maps were queried from Google Static Map APIs (http://code.google.com/apis/maps/) using the Get_google_map mapping package version 1.4 (https://uk.mathworks.com/matlabcentral/fileexchange/24113-get-google-map)

Supplementary Media 2 | Animation of the Internal Waves generated by the model is shown with the evolution of Non-Hydrostatic Pressure Potential normalised by density ($P_{NH}\cdot\rho^{-1}$, $m^2 \cdot s^{-2}$) over two tidal cycles. Mooring locations are shown with triangles (1-3), positions of vertical transects A-B and C-D and dissolved matter plume release sites (1-5, blue crosses) are also included. Media2_Internal_waves_NHPressure.gif 2.4Mb (https://figshare.com/s/519addf357f06e5fe693) Animation was generated using MATLAB R2015b (http://www.mathworks.com/).

Supplementary Media 3 | Animation shows a particulate matter plume spreading over bathymetry (lines, 50 m increment) during 10 days of model simulation. Plume contains $5.7\cdot10^5$ individual suspended particles in water (red) and settled (colours, m) on the seafloor. Points along the nodule collector tracks were aligned with equally-spaced Archimedes spiral, and shown on dashed in-cut to indicate the scale of the harvested zone during the last day (red) and since the beginning of experiment (green). Media3_2D_plume_movie.gif 1.6Mb (https://figshare.com/s/abd96d10a22263c42e3b) Animation was generated using MATLAB R2015b (http://www.mathworks.com/).

Supplementary Media 4 | Animation shows a particulate matter plume spreading over 3D bathymetry (lines, 50 m increment) during 10 days of model simulation. Plume contains $5.7\cdot10^5$ individual suspended particles in a water (grey) and settled (colours, m) on the seafloor. Media4_3D_plume_movie.gif 0.8Mb (https://figshare.com/s/b1056ea5d9570d9c654e) Animation was generated using MATLAB R2015b (http://www.mathworks.com/).

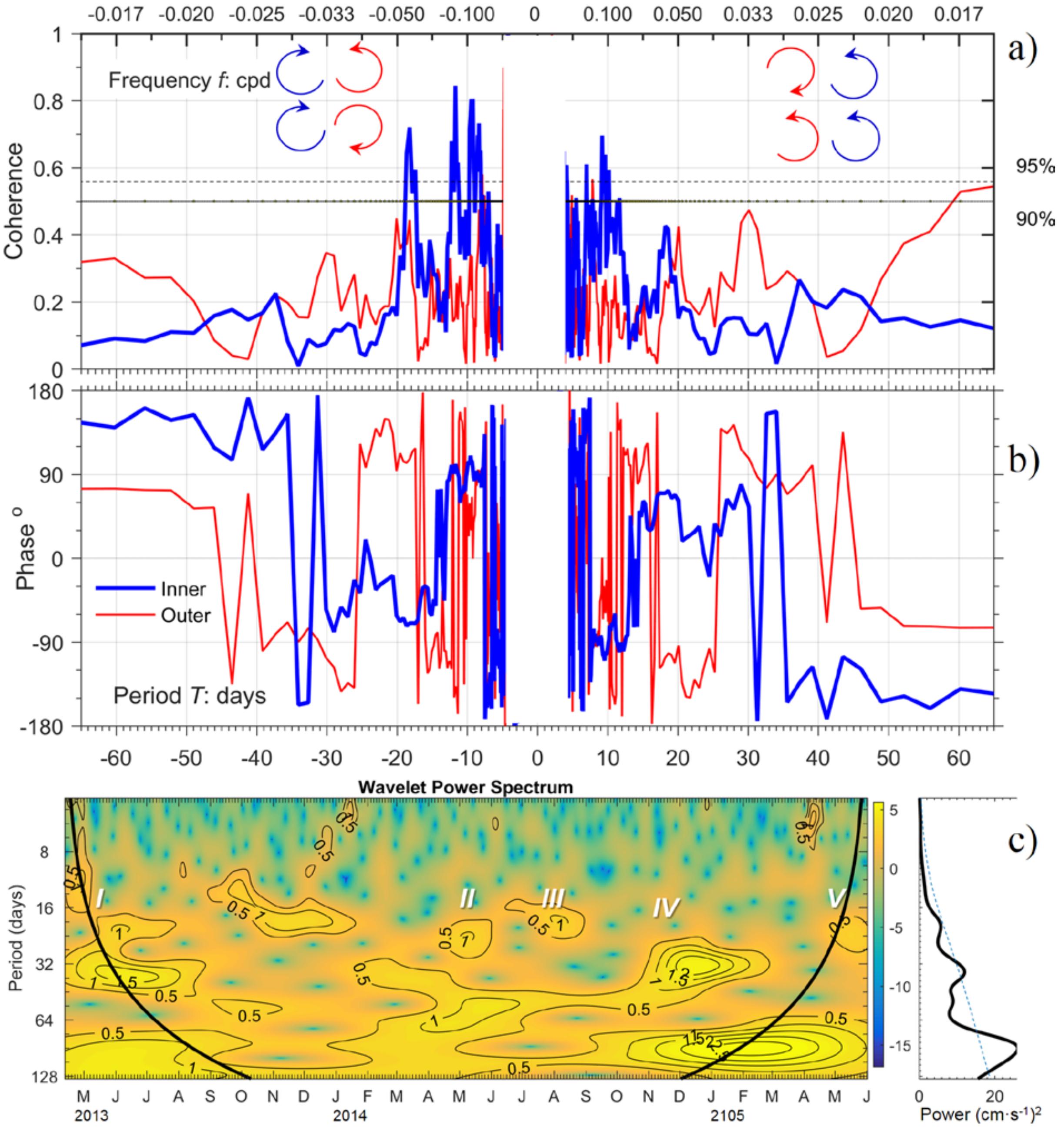

Supplementary Figure S1 | a-b, Complex Rotary Co-rotation (inner, thick blue) and Counter-rotation (outer, thin red) Coherence (**a**) and their Phases (**b**) were calculated for a period 2013.04.13 - 2015.06.01 between the surface geostrophic velocity (MADT-uv AVISO) and residual 24h averaged currents at 20 mab at mooring site 2. Dashed lines indicate 90% and 95% confidence levels, frequency axis *f:cpd* (cycles per day) is shown on top, and the noisy high-frequency segment (f>0.2 days^{-1}) is removed for clarity. **c**, Wavelet (Morlet) power spectrum of the seabed daily-averaged residual current speed normalised by its standard deviation. Thin contours show normalized variances of 0.5,1…2.5. Thick black line encloses regions of greater than 95% confidence level for a red-noise process with a lag-1 coefficient of 0.72. Edge effects are higher in both regions outside the 'cone of influence'. Latin numbers indicate eddies propagation time. High_Res_FS1 (https://figshare.com/s/f33be1f71cf3767ee962) Figure was plotted using MATLAB R2015b (http://www.mathworks.com/).

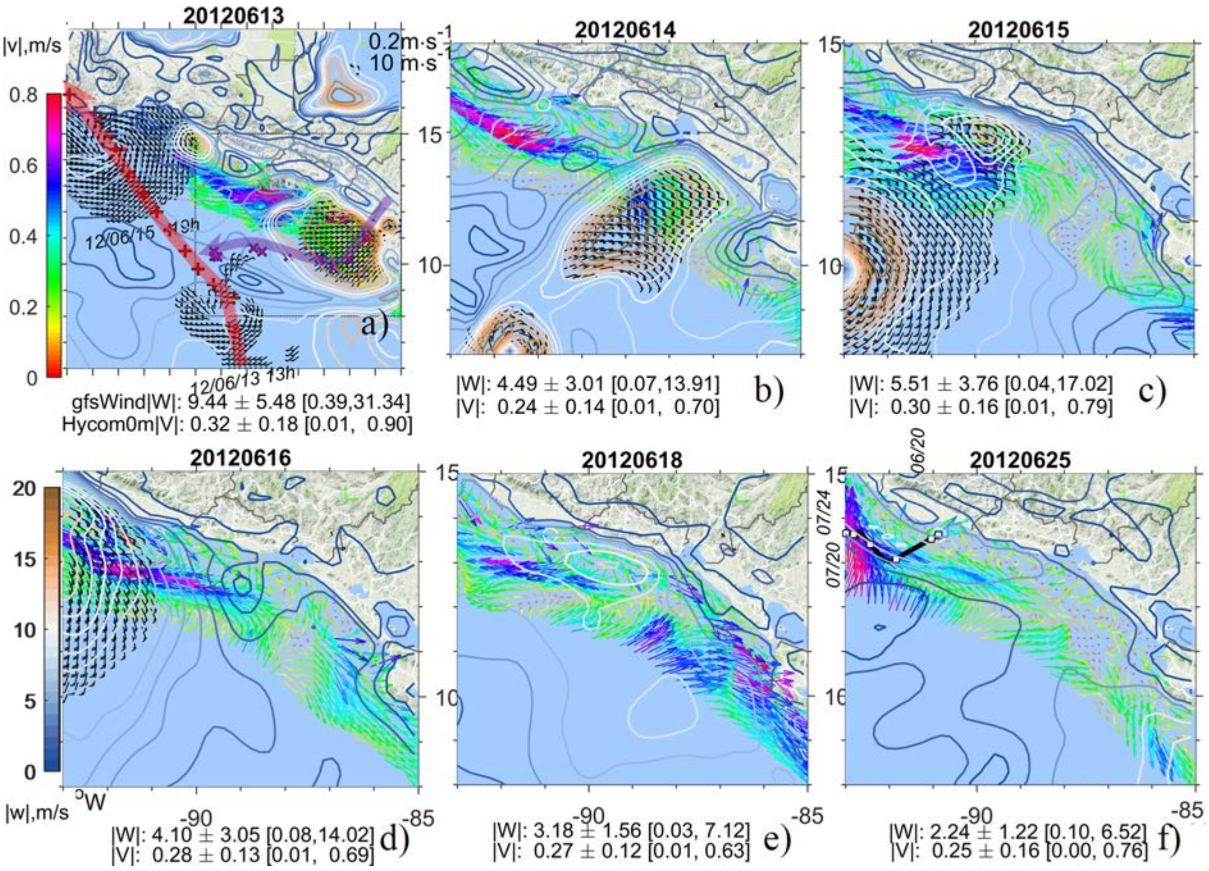

Supplementary Figure S2 | a-f, Wind direction (barbs >8 $m \cdot s^{-1}$ only are shown) and strength (|w|, blue-brown isotachs) at 10 m height (NCEP GFS[59]) overlaid with daily-averaged sea surface currents (v, coloured vectors, HYCOM[58]) in the area of Eddy Ia formation, indicated by white circle at each plot. High Papagayo wind patch propagation is shown with × magenta crosses every 6-hours until merged with the other tropical cyclone to ignite hurricane Carlotta on 14th June 2012, whose track[64] is shown by the red plus sign + (**a**). The Eddy Ia core daily track (black line with white circles) since 18th June 2012 was derived from 0.25^{o} SSH AVISO[31] maps (**f**). Freely-propagating, intra-seasonal and intra-annual equatorial Kelvin waves could be transformed into coastally-trapped waves (CTWs) on their arrival at the Central American coast and become amplified with poleward propagation along the coastal waveguide[45]. CTWs may then induce eddy shedding from the narrow coastal current as shown here. The vertical axis tilt inside such an eddy core is a signature of baroclinic instability due to vortex compression and stretching over varying bathymetry (*ibid*). High_Res_FS2 (https://figshare.com/s/e72eb6d311500a81c149). Figure was plotted using MATLAB R2015b (http://www.mathworks.com/).The maps on this figure were queried from Google Static Map APIs (http://code.google.com/apis/maps/) using the Get_google_map mapping package version 1.4 (https://uk.mathworks.com/matlabcentral/fileexchange/24113-get-google-map).

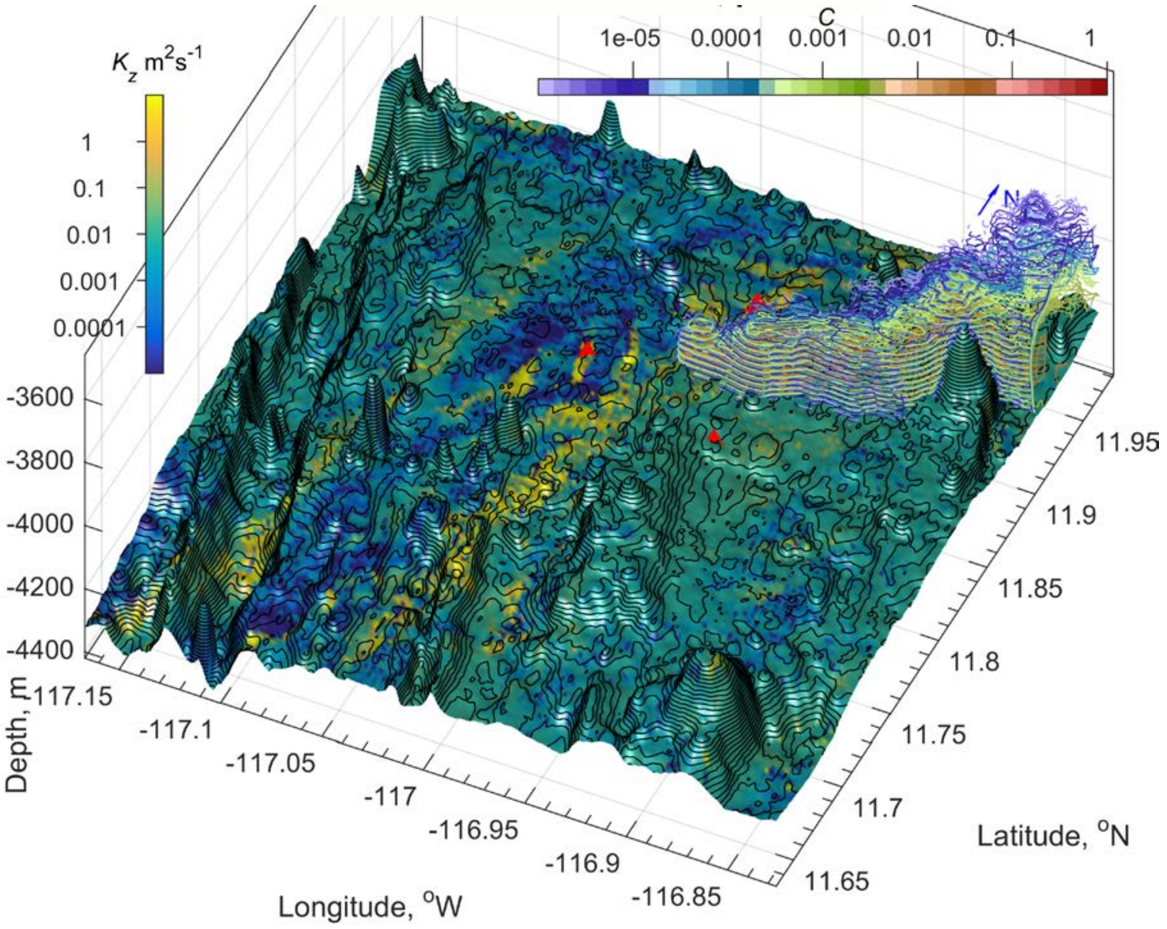

Supplementary Figure S3 | Neutrally buoyant plume spreading pattern (colours indicate tracer concentration *C* in fraction of 1) over 3D bathymetry (black lines, 50 m increment) overlaid with vertical mixing coefficients at the seabed (K_z, m^2·s^{-1}) on 30th April 2013, near-field model experiment I. Moorings location shown by small red triangles. High_Res_FS3. (https://figshare.com/s/17ecf9d3f778931e791c). Figure was plotted using MATLAB R2015b (http://www.mathworks.com/).

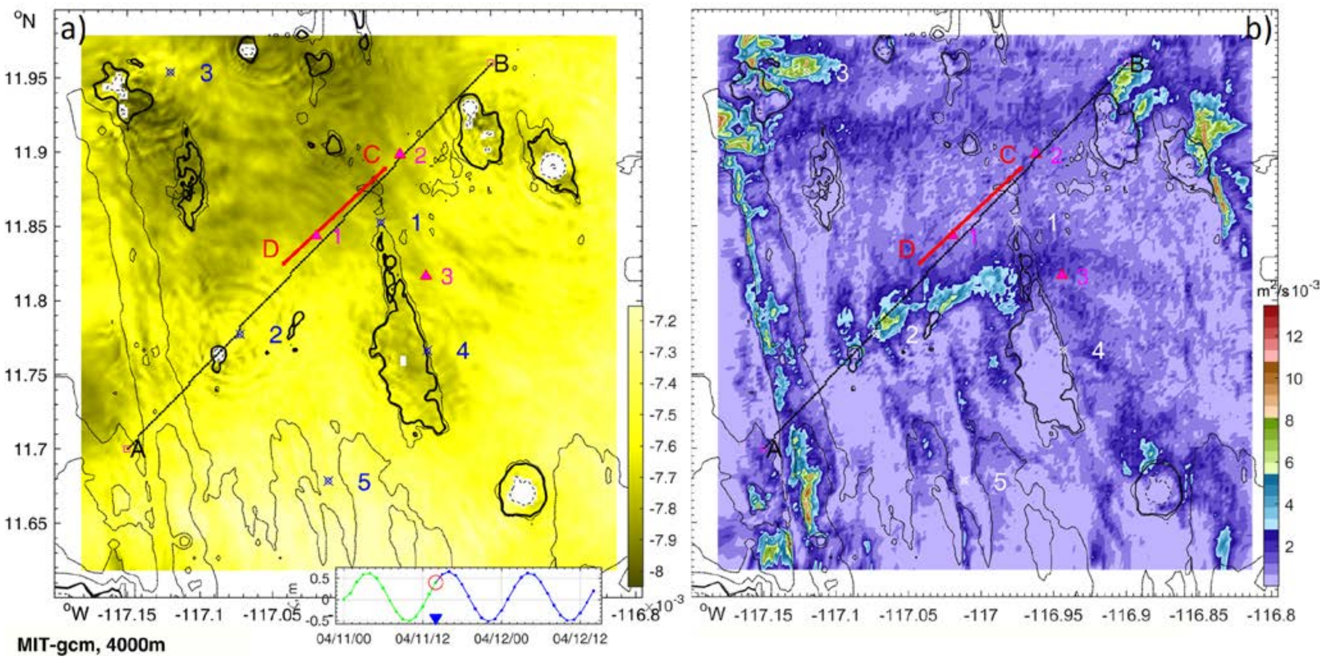

Supplementary Figure S4 | a, Modeled non-hydrostatic pressure potential (P_{NH}/ρ, $m^2 \cdot s^{-2}$, shadow) indicates radiation of Internal Waves from topography. **b**, Mixing *hotspots* are shown with the vertical diffusivity (10^{-3} $m^2 \cdot s^{-1}$) in layer 100 mab averaged over a month. High_Res_FS4 (https://figshare.com/s/2bcd0b2ddef67fb256c7). Animation (**a**) is available on-line: Internal_waves_NHPressure.gif (Media 2).Figure and aniamtion were plotted using MATLAB R2015b (http://www.mathworks.com/).

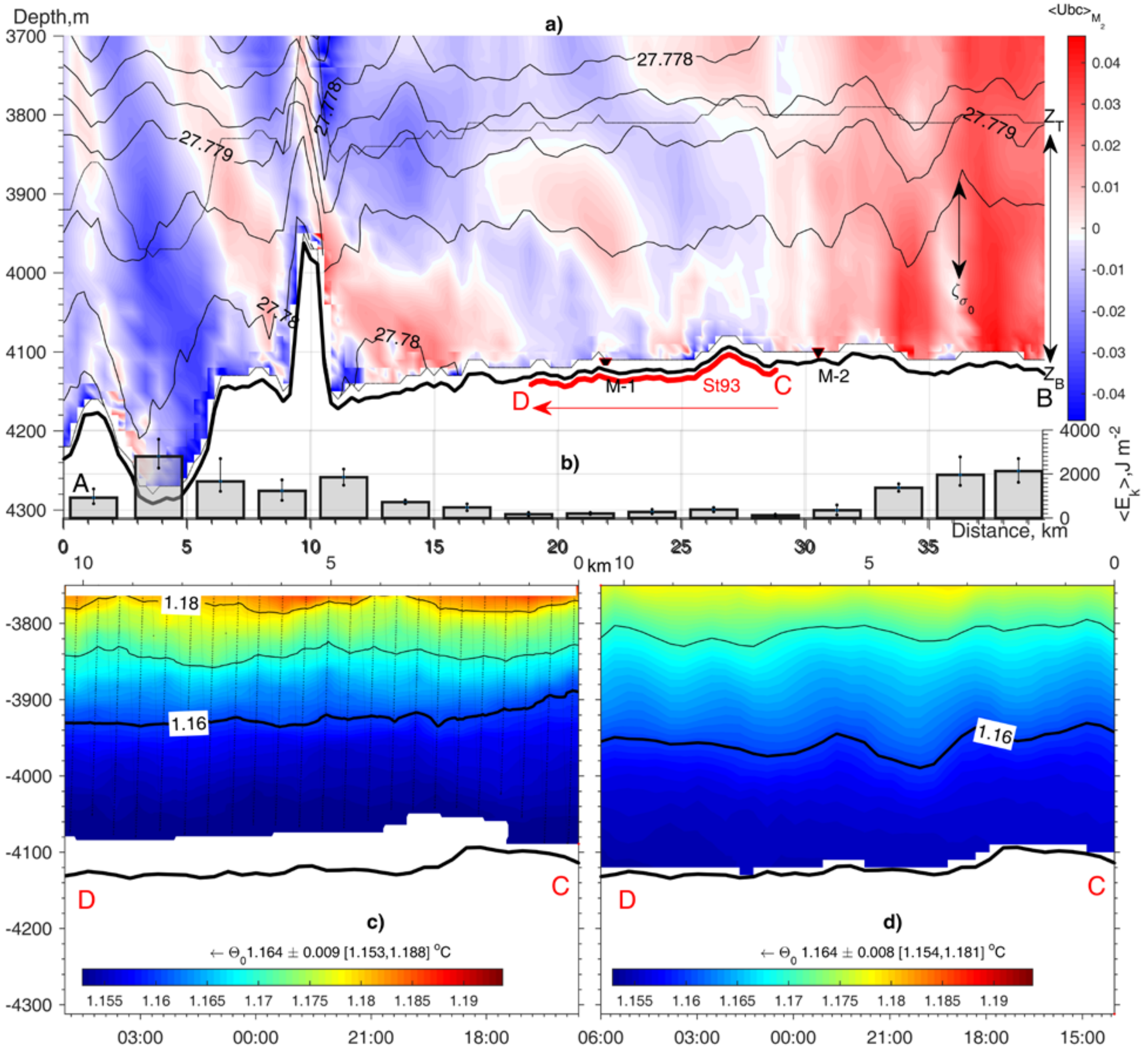

Supplementary Figure S5 | a, Typical vertical distribution of modelled horizontal current velocity (colours) overlaid with potential density isopycnals (black lines) along the transect A-B in Supplementary Figure S4. The range of isopycnal vertical displacements (ξ) is shown by the black double arrow. **b,** Grey blocks shows the values of Kinetic Energy and their standard deviations (bars) integrated over 2.5 km segments and within a layer 400 mab. **c,** Potential temperature (θ_0) distribution in a bottom boundary layer along the transect CD (red line) recorded by deep two-yo CTD Station 93 between points C (13:35, 4th June 2015, 11°53.850'N, 116°57.783'W, 4101 m) and D (6:31, 5th June, 11°49.089'N, 117°02.971' W, 4132 m) during a single tidal cycle[61]. **d,** θ_0 distribution along the same line according to the model, forced with the averaged CTD profiles collected in April 2013; note rise in thermal stratification over two years. High_Res_FS5 (https://figshare.com/s/3b2bdd2c7036a4b22219) Figure was plotted using MATLAB R2015b (http://www.mathworks.com/).

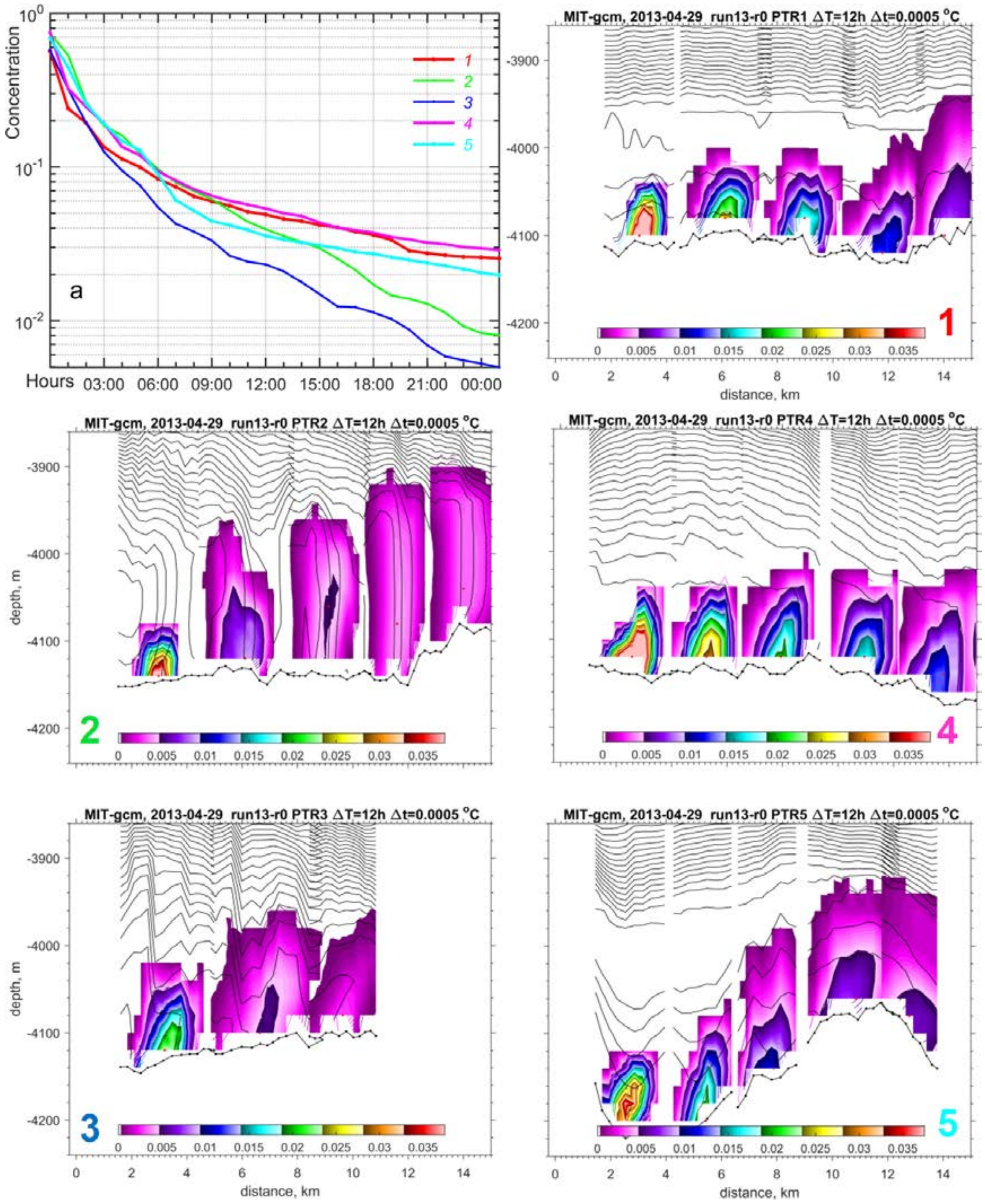

Supplementary Figure S6 | a, Modelled tracer concentration (parts of unit) dilution in plume core when tracers are injected close to the seabed in calm areas (**1**,**4**,**5**) and near mixing *hotspots* (**2**,**3**). Panels' **1-5** show vertical transects of the tracer concentration across the plume core (dotted lines on Fig. **4b**) and subsampled in 12 hour intervals. Bathymetry is shown by the dotted black lines every 100 m and potential isotherms are shown by thin lines with interval 0.0005°C. High_Res_FS6 (https://figshare.com/s/553624f0340dcbf34f3c) Figure was plotted using MATLAB R2015b (http://www.mathworks.com/).

Supplementary Table S1 | Parameters of mesoscale eddies tracked from Papagayo (Ia) and Tehuantepec (Ib-V) in a period from June 2012 until May 2015: dates at origin and destination locations, drift duration, mean, maximum speed and its standard deviation, hurricanes names, dates and maximum wind speed.

ID	At origin			At destination			N	Mean	STD	Pick	Hurricane	Date	Wind
	Date	Lon	Lat	Lon	Lat	Date		$\lvert V \rvert$	σ_V	V	name		speed
#	yymmdd	°W	°N	°W	°N	yymmdd	days	$m \cdot s^{-1}$	$m \cdot s^{-1}$	$m \cdot s^{-1}$		yymmdd-dd	$km \cdot h^{-1}$
I_a	120618	90.875	13.625	116.9952	11.2487	130329	284	0.14	0.06	0.30	Carlotta	120612-16	175
I_b	120916	95.50	15.50	99.00	12.50	121030	44	0.16	0.05	0.30	Kristy	120912-17	95
II	131020	96.75	14.75	117.5763	11.2128	140420	182	0.16	0.06	0.33	Raymond	131018-30	205
III	131102	95.70	14.95	116.6564	11.0477	140706	246	0.13	0.05	0.30	Sonia	131101-04	75
IV	131225	96.25	14.95	117.0297	11.8435	141120	330	0.11	0.08	0.89			
V	141008	95.50	15.50	116.1081	10.8378	150412	186	0.15	0.04	0.26	Simon	141001-07	215
mean				nearest to moorings BGR-site			**246**	**0.14**	**0.06**	**0.40**			

Supplementary Table S2 | Statistical parameters of high-resolution MIT-gcm model performance skills against velocity measurements in a layer 20 mab (4100 m) at three mooring sites during 38 days in the period 2013.04.12 - 2013.05.20: hourly sub-sampled (zonal *u*, meridional *v* currents speed, $cm \cdot s^{-1}$) and residual (de-tided 12-h averaged $\langle u \rangle_d, \langle v \rangle_d$). RMSD and RMSE are the root mean square difference and error respectively and $\boldsymbol{I_A}$ is Wilmott' Index of Agreement[71] defined as:

$$I_A = 1 - \frac{\sum_{i=1}^{N}\left(M_i - O_i\right)^2}{\sum_{i=1}^{N}\left(\left|M_i - \bar{O}\right| + \left|O_i - \bar{O}\right|\right)^2}$$

, where $\boldsymbol{M}$ and $\boldsymbol{O}$ are the modelled and observed variables, $\bar{\boldsymbol{M}}$ and $\bar{\boldsymbol{O}}$ are the whole timeseries mean, $\boldsymbol{\sigma_m}$ and $\boldsymbol{\sigma_o}$ are the standard deviations.

Mooring N^o		1				2				3			
Parameters		*u*	*v*	$\langle u \rangle_d$	$\langle v \rangle_d$	*u*	*v*	$\langle u \rangle_d$	$\langle v \rangle_d$	*u*	*v*	$\langle u \rangle_d$	$\langle v \rangle_d$
Observations	$\bar{O}$	3.55	4.11	3.54	4.14	5.29	3.26	5.31	3.27	4.11	1.81	4.10	1.83
Obs. STD	σ_o	2.69	2.53	2.53	2.11	2.74	2.27	2.46	1.87	3.82	2.38	3.54	1.85
Model	$\bar{M}$	3.23	2.00	3.23	1.99	4.18	0.94	4.24	0.90	4.57	-0.60	4.56	-0.63
Model STD	σ_m	3.26	3.73	2.94	3.48	2.29	3.05	1.87	2.51	3.12	2.07	2.81	1.54
Correlation	R	0.83	0.71	0.93	0.80	0.74	0.79	0.87	0.93	0.87	0.40	0.94	0.65
RMSD	D	1.83	2.62	1.11	2.18	1.84	1.87	1.22	1.00	1.90	2.45	1.30	1.43
RMSE	E	1.85	3.36	1.16	3.06	2.15	2.98	1.63	2.58	1.96	3.43	1.38	2.84
Taylor Score[72]	S	0.67	0.46	0.84	0.51	0.56	0.59	0.71	0.80	0.73	0.24	0.84	0.45
Index of Agreement[71]	I_A	0.90	0.72	0.95	0.72	0.81	0.73	0.86	0.73	0.92	0.54	0.95	0.60

[71] Wilmott CJ. Some comments on the evaluation of model performance. *Bull American Meteorol Society* 1982, **63:** 1309-1313.

[72] Taylor K. Summarizing multiple aspects of model performance in a single diagram. *Journal of Geophysical Res* 2001, **106**(D7)**:** 7183–7192.

Supplementary Table S3 | Annual settling flux (ASF) and the thickness of settled SPM layer calculated over periods of 10 days and 1000 years in numerical Experiment III and measured in the Eastern Tropical Pacific; symbols refer to **Figure 6**.

N°	Sample Depth	Annual Settling Flux	Thickness	Thickness	Distance to source	Longitude	Latitude	Duration	Date	Source
	m	$g \cdot m^{-2} \cdot yr^{-1}$	$mm \cdot 10\ days^{-1}$	$mm \cdot k \cdot yr^{-1}$	km	°W	°N	days	yyyy.dd.mm	
○ 1	4150	150,221	51.45	187,777	0	-117.0750	11.7800	10	2013.04.22	*
2	4162	197,969	67.80	247,461	0.4	-117.0768	11.7764		2013.05.02	
3	4169	43,769	14.99	54,711	1	-117.0823	11.7764			
4	4143	202	0.07	253	3	-117.0768	11.8088			
5	4126	1,806	0.62	2,257	5	-117.0934	11.8232			
6	4128	2.9	0.001	3.6	10	-117.0143	11.8502			
7	4130	138	0.05	173	15	-117.1228	11.9078			
▼ A	4025	4.80	0.00016	6.0	20.7	-117.0070	11.9537		2010.05.-	†
B	4106	2.80	0.00010	3.5	22.1	-116.9767	11.9538		2010.05.-	
■ a	2603	3.27	0.00011	4.1	JGOFS‡	-103.90	12.83	222	1981.05.23	§
b	5582	4.05	0.00014	5.1		-151.48	15.35	98	1978.07.01	**
c	3660	6.64	0.00023	8.3		-117.0	17.5	24	1982.12.01	††
d	3400	27.34	0.00094	34.2		-140.0	11.0	100	1983.04.08	‡‡
e	3400	6.36	0.00022	8.0		-140.0	11.0	112	1983.10.25	§§
■	4880	30,737	10.53	38,421	0.05	-128.6666	12.8833	19	1993.08.14	***
	4890	46	0.016	58	6.00	-128.6396	12.8473	19	1993.09.02	
♦ 1	4842	2,278	0.78	2,848	0.50	-128.7114	12.9389	Scale factor R=31.8 applied, Equivalent thickness, $mm\ 10\ days^{-1}$	◊ 24.9	†††
2	4843	3,072	1.05	3,840	0.27	-128.7066	12.9277		33.5	
3	4851	3,412	1.17	4,265	0.25	-128.7102	12.9335		37.2	
4	4860	2,980	1.02	3,724	0.28	-128.7132	12.9391		32.5	
5	4858	4,028	1.38	5,036	0.14	-128.7058	12.9245		44.0	
6	4859	3,619	1.24	4,524	0.11	-128.7157	12.9411		39.5	
7	4846	2,280	0.78	2,850	0.07	-128.7068	12.9236		24.9	
8	4906	438	0.15	548	0.27	-128.7104	12.9270		4.8	
9	4858	607	0.21	759	0.19	-128.7170	12.9394		6.6	
10	4870	815	0.28	1,018	0.20	-128.7088	12.9253		8.9	
11	4837	924	0.32	1,155	0.18	-128.7121	12.9311		10.1	
12	4833	638	0.22	797	0.22	-128.7153	12.9362		7.0	
14	4864	44	0.015	55	0.37	-128.7149	12.9333		0.5	
15	4842	2,376	0.81	2,970	0.43	-128.7075	12.9314		25.9	

* SPM numerical Experiment III, with kl10 mixing scheme[68], this study.
† Sediment cores at two stations A5 at Eastern CCZ BGR licence area[51].
‡ Global sediment traps data portal[78] http://usjgofs.whoi.edu/mzweb/data/Honjo/sed_traps.html
§ *ibid*[78], http://usjgofs.whoi.edu/jg/serv/jgofs/SMP_results/sed_traps_ann_flux.html4 ?Project_ID %20contains%20PARFLUX-Clipperton_Fracture_Zone
** *ibid* [77], http://usjgofs.whoi.edu/jg/serv/jgofs/SMP_results/sed_traps_ann_flux.html4?Project_ID%20contains%20 PARFLUX-East_Hawaii_Abyssal_Plain-Site_P1
†† *ibid* [79],http://usjgofs.whoi.edu/jg/serv/jgofs/SMP_results/sed_traps_ann_flux.html4?Project_ID %20contains%20EP11
‡‡ *ibid* [80] cap 3 (el Nino), jgofs.whoi.edu/jg/serv/jgofs/SMP_results/sed_traps_ann_flux.html4?Project_ID %20contains%20MANOP-Site-S
§§ *ibid* [80] cap 4, MANOP-Site-S
*** Visual near- and far-field data (photo), Benthic Impact Experiment (BIE) [50,51]
††† Sediment traps content, *ibid* [51].
