# Supplementary figures and images for: Impact of remotely generated eddies on plume dispersion at abyssal mining sites in the Pacific
Source: Sci Rep. 2017 Dec 5;7:16959. doi: 10.1038/s41598-017-16912-2 (PMC5717004; doi:10.1038/s41598-017-16912-2)

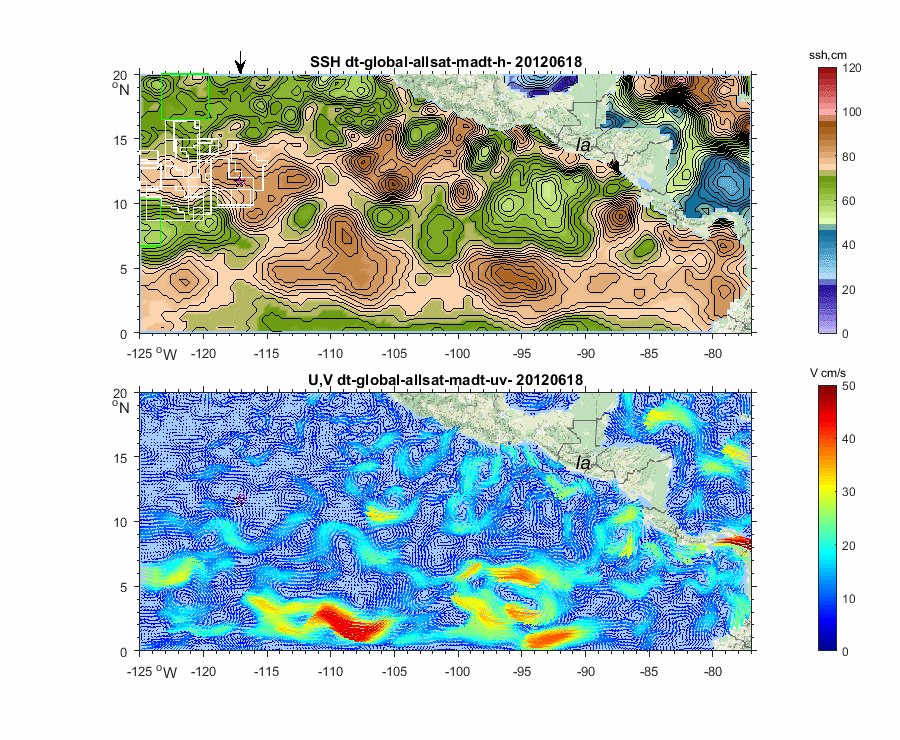

Supplement: Supplementary file 2 — Supplementary Media 1 [file 41598_2017_16912_MOESM2_ESM.gif]

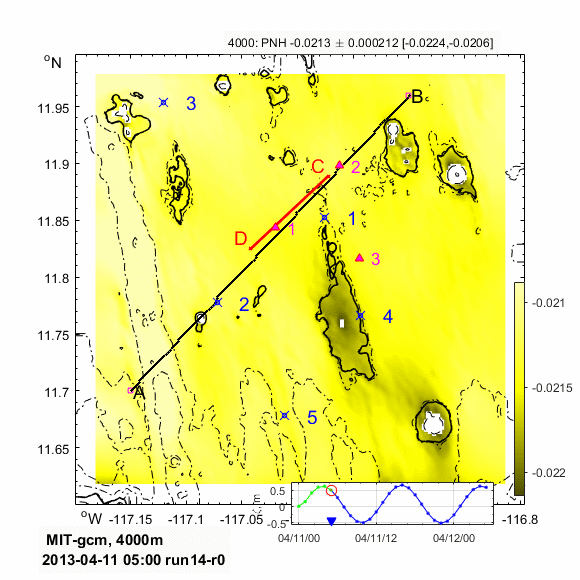

Supplement: Supplementary file 3 — Supplementary Media 2 [file 41598_2017_16912_MOESM3_ESM.gif]

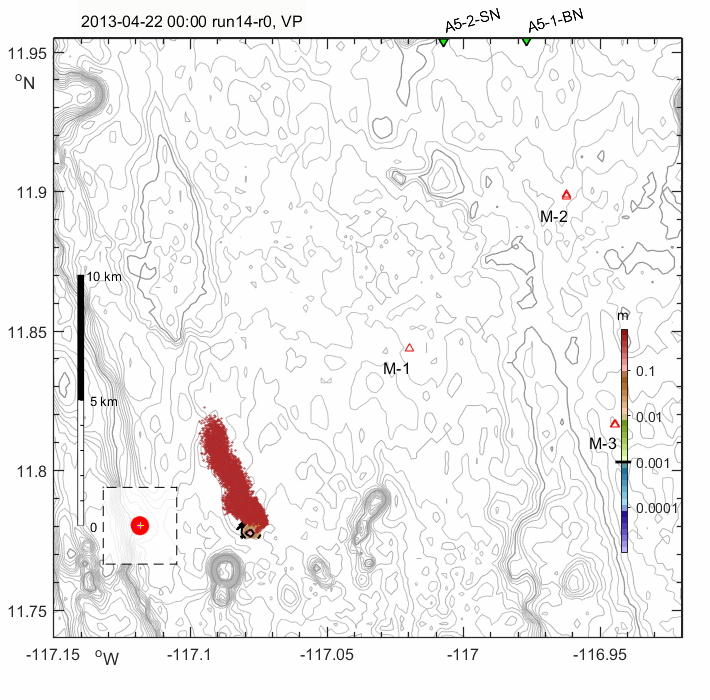

Supplement: Supplementary file 4 — Supplementary Media 3 [file 41598_2017_16912_MOESM4_ESM.gif]

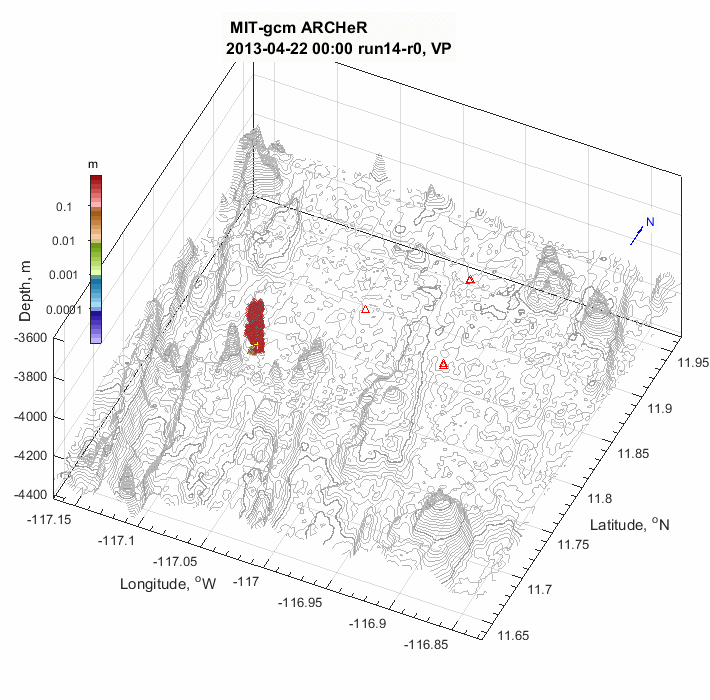

Supplement: Supplementary file 5 — Supplementary Media 4 [file 41598_2017_16912_MOESM5_ESM.gif]
